# Restoration of energy homeostasis under oxidative stress: Duo synergistic AMPK pathways regulating arginine kinases

**DOI:** 10.1371/journal.pgen.1010843

**Published:** 2023-08-03

**Authors:** Nan Zhang, Xiangkun Meng, Heng Jiang, Huichen Ge, Kun Qian, Yang Zheng, Yoonseong Park, Jianjun Wang

**Affiliations:** 1 College of Plant Protection, Yangzhou University, Yangzhou, China; 2 Jiangsu Lixiahe Institute of Agricultural Sciences, Yangzhou, China; 3 Department of Entomology, Kansas State University, Manhattan, Kansas, United States of America; The University of North Carolina at Chapel Hill, UNITED STATES

## Abstract

Rapid depletion of cellular ATP can occur by oxidative stress induced by reactive oxygen species (ROS). Maintaining energy homeostasis requires the key molecular components AMP-activated protein kinase (AMPK) and arginine kinase (AK), an invertebrate orthologue of the mammalian creatine kinase (CK). Here, we deciphered two independent and synergistic pathways of AMPK acting on AK by using the beetle *Tribolium castaneum* as a model system. First, AMPK acts on transcriptional factor forkhead box O (FOXO) leading to phosphorylation and nuclear translocation of the FOXO. The phospho-FOXO directly promotes the expression of AK upon oxidative stress. Concomitantly, AMPK directly phosphorylates the AK to switch the direction of enzymatic catalysis for rapid production of ATP from the phosphoarginine-arginine pool. Further *in vitro* assays revealed that Sf9 cells expressing phospho-deficient AK mutants displayed the lower ATP/ADP ratio and cell viability under paraquat-induced oxidative stress conditions when compared with Sf9 cells expressing wild-type AKs. Additionally, the AMPK-FOXO-CK pathway is also involved in the restoration of ATP homeostasis under oxidative stress in mammalian HEK293 cells. Overall, we provide evidence that two distinct AMPK-AK pathways, transcriptional and post-translational regulations, are coherent responders to acute oxidative stresses and distinguished from classical AMPK-mediated long-term metabolic adaptations to energy challenge.

## Author summary

AMP-activated protein kinase (AMPK) and phosphoarginine-arginine kinase (AK) shuttle system play essential roles in regulating the cellular and organismal energy homeostasis under various stresses in invertebrates, although the regulatory mechanisms culminating in the ATP homeostasis remain largely unknown. In this work, using the red flour beetle, *Tribolium castaneum*, as a model organism, we found that AMPK upregulates the expressions of *AKs* upon oxidative stress through direct phosphorylation of transcriptional factor forkhead box O (FOXO). The phosphorylation of FOXO followed by the nuclear translocation directly enhances the transcription levels of *AK1* and *AK2*. At the same time, the AMPK directly phosphorylates AKs, resulting in a shift of the AK-catalyzed reversible reaction toward the direction of ATP generation. Taken together, our research presents two parallel synergistic AMPK-AK pathways that protect the cells from rapid depletion of ATP under acute oxidative stress condition, which is distinguished from classical AMPK-mediated long-term metabolic adaptations to energy challenge.

## Introduction

Energy homeostasis is a crucial requirement for living cells in response to nutritional and environmental stresses. The evolutionarily conserved AMP-activated protein kinase (AMPK) functions as a sensor measuring cellular energy status, as well as other signals such as nutrient availability [1]. The heterotrimeric AMPK complex consists of an alpha catalytic subunit (α) containing protein kinase catalytic domain, regulatory gamma subunits (γ) sensing AMP:ADP:ATP ratio, and a non-catalytic beta subunit (β). Increased AMP or ADP over ATP sensed by the γ subunit promotes the phosphorylation of the α subunit and protects it from dephosphorylation [2–5]. An alternative nucleotide-independent AMPK activation pathway is triggered by increases in intracellular Ca^2+^ levels [6]. The activated AMPK phosphorylates enzymes involved in metabolic pathways, leading to the stimulation of catabolic reactions that generate ATP and inhibition of anabolic processes that consume ATP [7–9]. Additionally, AMPK can also contribute to energy balance via transcriptional regulation of metabolic enzymes in the long term, although its downstream transcriptional pathways remain largely elusive [10].

There are emerging studies indicating that AMPK not only functions in the regulation of energy metabolism but is also involved in the response to oxidative stress. Reactive oxygen species (ROS), generated from xenobiotic biotransformation or the process of fuel oxidation, can disrupt the cellular redox homeostasis and trigger oxidative stress by directly oxidizing and damaging DNA, proteins, and lipids. ROS can modulate AMPK activity by direct posttranslational modification [11,12], or indirectly activate AMPK by increasing cellular AMP and/or ADP levels [13]. In mammals, the deletion of AMPK α1 significantly increased the levels of ROS and oxidized proteins in murine erythrocytes [14], and over-expression of AMPK in human embryonic kidney cells (HEK293) protected H_2_O_2_-induced cell damage [15]. In *Drosophila melanogaster*, inhibition of dAMPKα in muscle enhances sensitivity to paraquat-induced oxidative stress [16]. Nevertheless, the underlying mechanisms of AMPK response to oxidative stress are not fully understood.

Another important component in cellular energy homeostasis and ROS response is the phosphagen kinase (PK) shuttle system as a temporal and spatial energy buffer during periods of high-energy demand or energy supply fluctuations [17,18]. During PK evolution, it has diverged into two distinct clusters: creatine kinase (CK), the PK exclusive in vertebrates, and arginine kinase (AK), the most widely distributed PK among invertebrates [19,20]. Therefore, insect AK is the counterpart of vertebrate CK. For energy metabolism, PK catalyzes the reversible transfer of the γ-phosphoryl group of ATP to the guanidine substrate, yielding ADP and high-energy phosphagen, a phosphorylated guanidine [17]. In addition, it has been demonstrated that activation of the creatine phosphate (CrP)/CK system causes a decrease in ROS generation [21–23]. Similarly, overexpressing AK showed significantly increased survival upon exposure to hydrogen peroxide in *Trypanosoma cruz*i [24] and the TbAK-depleted *Trypanosoma brucei* cell line became >400-fold more sensitive to hydrogen peroxide [25]. However, the regulatory mechanisms of CK and AK remain largely unknown.

In contrast to mammalian genome encoding multiple AMPK subunit isoforms (α1, α2, β1, β2, γ1, γ2, γ3), each AMPK subunit of insects is encoded by a single gene [26,27], which makes insects particularly appealing for studying the downstream targets of the AMPK signaling pathway. While a single AK gene was identified in most insect species, two AK genes were annotated in the genomes of *Anopheles gambiae*, *Aedesae gypti*, and *Apis mellifera* [28]. Recently, we have found that RNAi-mediated knockdown of *TcAMPKα*, as well as *TcAK1* and *TcAK2*, resulted in the increased sensitivity to paraquat-induced oxidative stress in the red flour beetle, *Tribolium castaneum* [26,29].

The similar function of AMPK and AK in the maintenance of energy homeostasis and protection against oxidative stress raises the possibility that these two factors are on the same pathway for orchestrating the responses. In the present study using *T*. *castaneum* as a model organism, we provided evidence that AMPK modulated the oxidative stress-induced expression of *AK1* and *AK2* via the transcriptional factor forkhead box O (FOXO) and changed the catalytic activity of AK1 and AK2 via direct phosphorylation. The results demonstrate that two independent AMPK to AK pathways synergize in maintaining ATP homeostasis under oxidative stress conditions.

## Results

### ATP levels under oxidative stress are maintained by AMPK, AK1 and AK2 in *T*. *castaneum*

**Fig 1:**
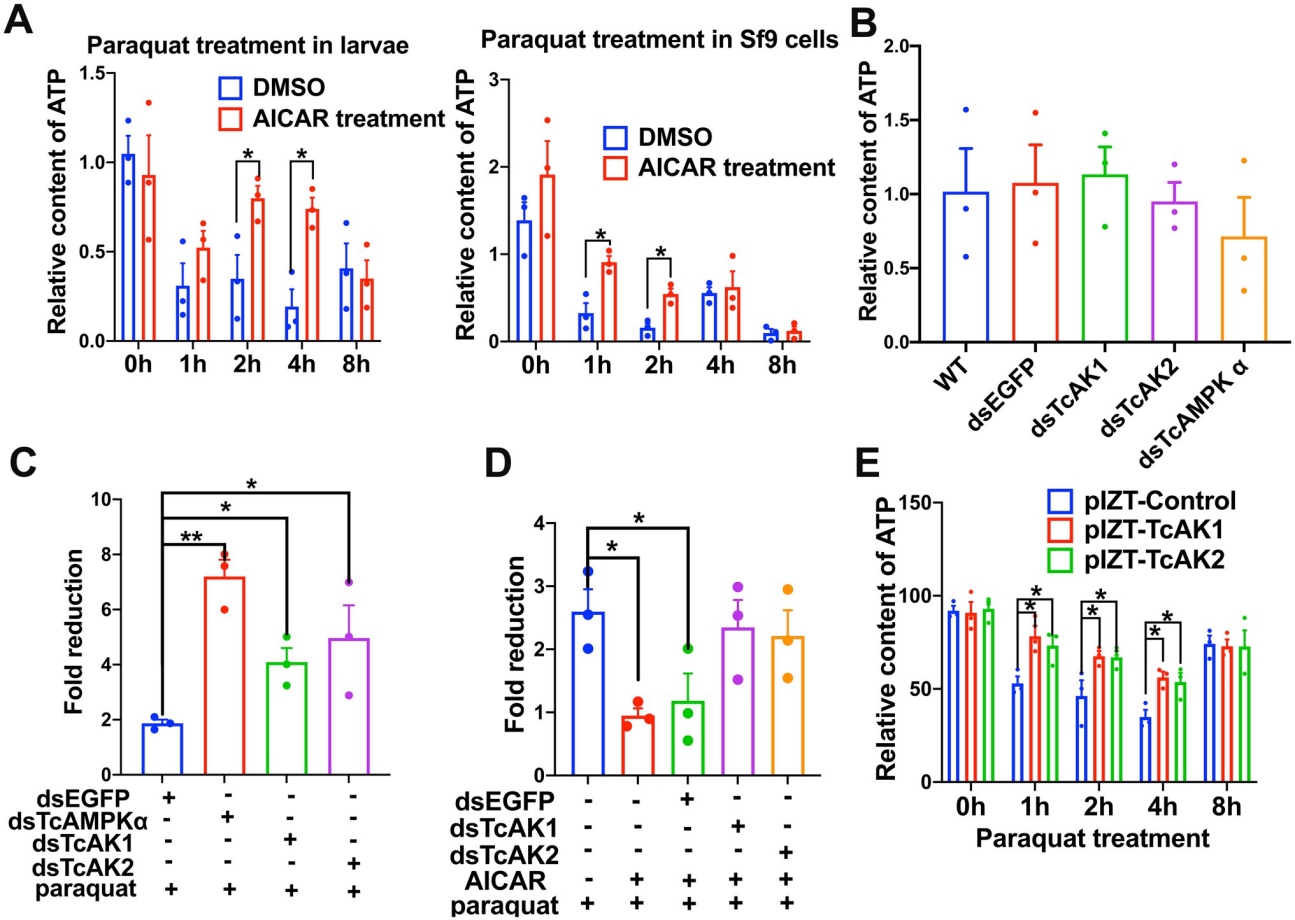
ATP homeostasis is maintained by TcAMPK, TcAK1 and TcAK2 under oxidative stress conditions. (a) AICAR treatment prevented paraquat-induced ATP depletion *in vivo* or *in vitro*. (b) Knockdown of *TcAK1*, *TcAK2*, or *TcAMPKα* mRNA expression did not affect the basal ATP content in beetles. (c) Knockdown of *TcAK1*, *TcAK2*, or *TcAMPKα* mRNA expression increased the fold of ATP decrease caused by paraquat treatment. (d) Knockdown of *TcAK1* or *TcAK2* mRNA expression attenuated the AICAR-induced increase in ATP content under paraquat treatment. Data were expressed as mean ± SEM (n = 3 biologically independent replicates). Asterisks indicate differences statistically significant at * *P* < 0.05 and ** *P* < 0.01 (student’s t-test).

The essential role of AMPK in maintaining cellular energy homeostasis was investigated by treatment with 5-aminoimidazole-4-carboxamide ribonucleotide (AICAR), an AMP analog well known as an AMPK activator [30]. ATP content at normal and oxidative stress conditions was further measured after RNAi of *TcAK1*, *TcAK2*, and *TcAMPKα*. Our results showed that AICAR treatment prevented the depletion of ATP caused by paraquat treatment in larvae of *Tribolium* and *Spodoptera frugiperda* (Sf9) cells (Fig 1A). Knockdown of the mRNA expression of *TcAK1*, *TcAK2*, and *TcAMPKα* significantly intensified paraquat-induced depletion of ATP compared with the negative control dsEGFP injection and WT groups, while they did not change the basal ATP content (Fig 1B and 1C). Moreover, knockdown of either *TcAK1* or *TcAK2* mRNA expression significantly reduced the AICAR-induced stability of ATP content under oxidative stress (Fig 1D), indicating the functional redundancy of the two AKs having 70.2% amino acid identity [29]. Thus, both AMPK and AKs were involved in the maintenance of ATP homeostasis under oxidative stress in *T*. *castaneum*.

### AMPK upregulates the transcript levels of *AK1* and *AK2* under oxidative stress

**Fig 2:**
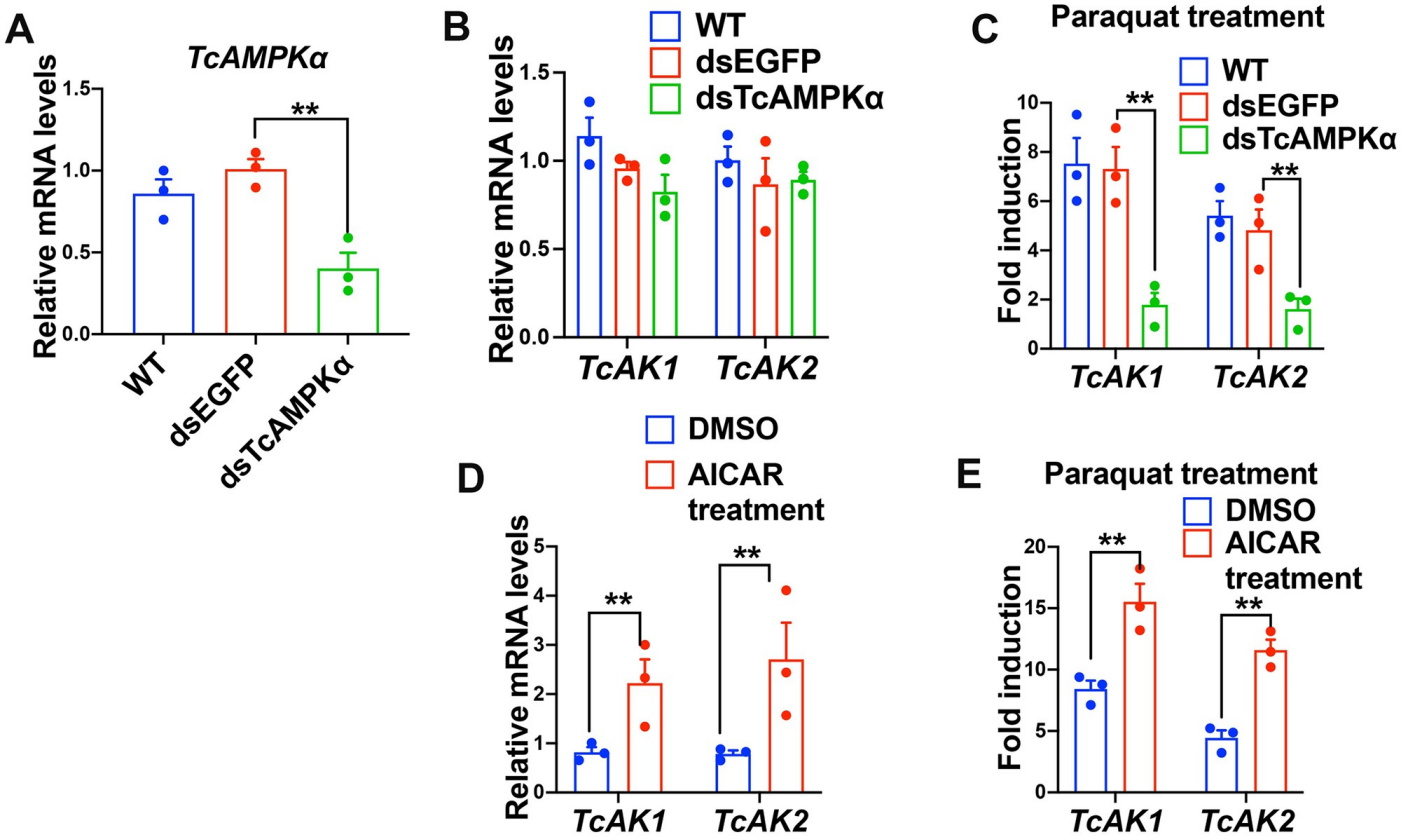
TcAMPKα is involved in the oxidative stress-induced expression of *TcAK1* and *TcAK2*. (a) The knockdown efficiency of *TcAMPKα* mRNA expression. (b) Knockdown of *TcAMPKα* mRNA expression did not affect the basal mRNA expression of *TcAK1* and *TcAK2*. (c) Knockdown of *TcAMPKα* mRNA expression attenuated the paraquat-induced mRNA expression of *TcAK1* and *TcAK2*. (d) AICAR treatment increased the basal mRNA expression of *TcAK1* and *TcAK2*. (e) AICAR treatment increased the paraquat-induced expression of *TcAK1* and *TcAK2*. mRNA expression was determined by qPCR using ribosomal protein S3 (rps3) as an internal reference. Data are expressed as mean ± SEM (n = 3 biologically independent replicates). Asterisks indicate differences statistically significant at **P< 0.01 (student’s t-test).

To investigate whether AMPK regulates ATP homeostasis through AKs under oxidative stress, we first examined the transcriptional regulation of AKs by AMPK. Suppression of *TcAMPKα* by RNAi was followed by measurements of basal and oxidative stress-induced expression of *TcAKs*. Compared with the dsEGFP-injected beetles, the mRNA level of *TcAMPKα* was reduced to 60.2% on the fourth day after the injection of dsTcAMPK*α* into 15-day-old larvae (Fig 2A). There was no significant change in the basal mRNA expressions of *TcAK1* and *TcAK2* (Fig 2B), whereas knockdown of *TcAMPKα* significantly suppressed the induction of *TcAK1* and *TcAK2* after exposure to paraquat (Fig 2C). These results suggest that *TcAMPKα* is involved in the paraquat-induced expression of *TcAK1* and *TcAK2*.

As an independent assessment of TcAMPK-mediated *TcAK1* and *TcAK2* induction, we treated the beetles with AICAR and found significantly enhanced basal levels of mRNA expression for *TcAK1* and *TcAK2* (2.7 and 3.4 folds, respectively, Fig 2D) as well as paraquat-induced expression (1.8 and 2.6 folds, respectively, Fig 2E). The results indicate that activation of TcAMPK is the upstream signal for the oxidative stress-induced expression of *TcAKs*. Since AMPK activation has been reported to cause an induction in transcriptional activity of the stress-responsive transcriptional factor FOXO in mammals [31,32], we speculated that TcAMPK might be acting through FOXO signaling to modulate the transcription of *TcAKs*.

### Paraquat induces FOXO expression and nuclear translocation

**Fig 3:**
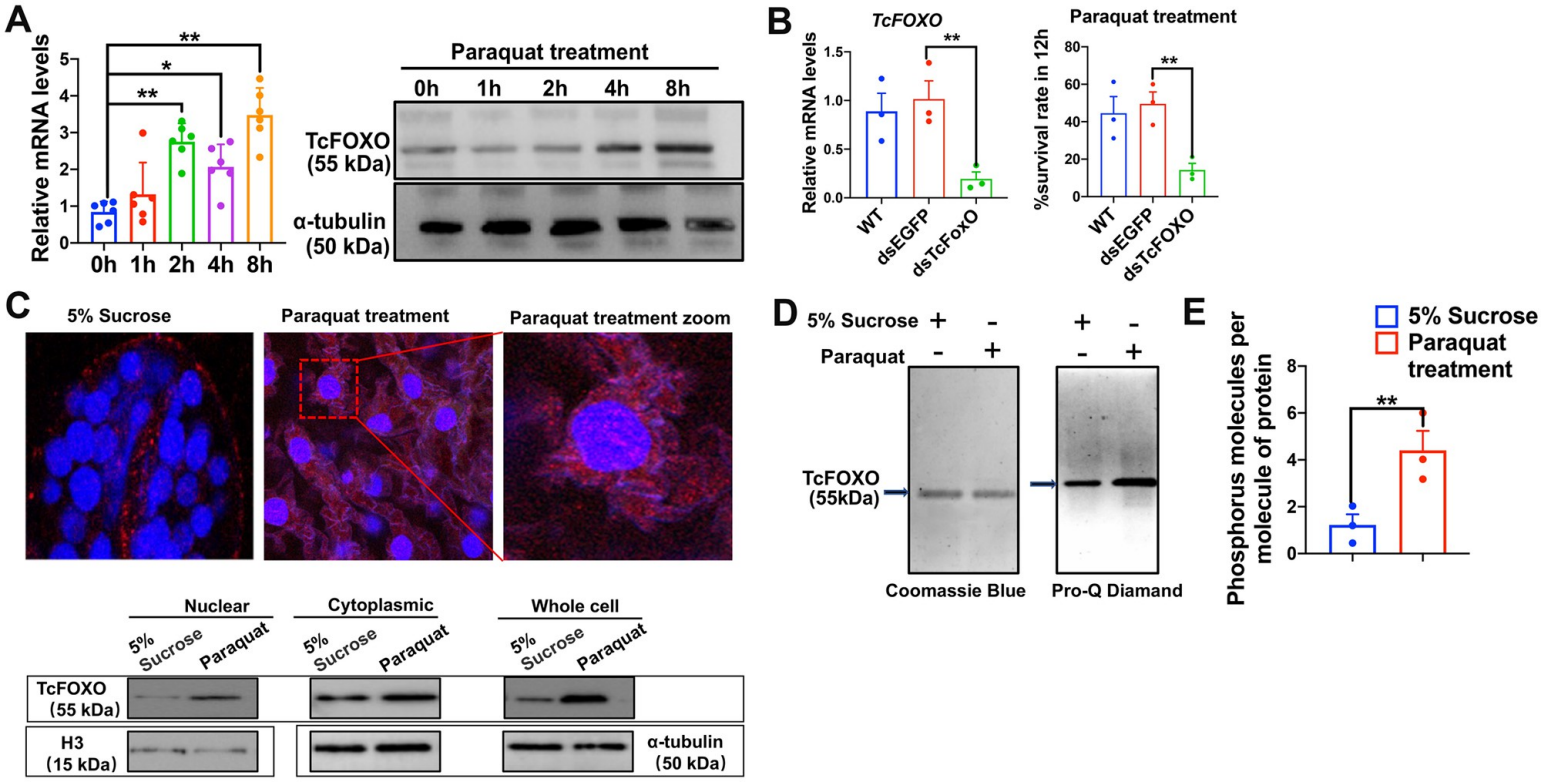
Oxidative stress promotes phosphorylation and nuclear translocation of TcFOXO. (a) Paraquat-induced mRNA and protein expression of TcFOXO at different times post-treatment. mRNA expression was determined by qPCR using ribosomal protein S3 (rps3) as an internal reference. Protein expression was determined by western blot using the anti-α-tubulin antibody as a loading control. These experiments were repeated three times with similar results. (b) The effects of knockdown of *TcFOXO* mRNA expression on tolerance of beetles to paraquat-induced oxidative stress. The larvae were collected for mRNA expression analysis and tolerance assay on the fourth day after dsTcFOXO treatment. The dsTcFOXO-injected beetles were treated with 20 mM paraquat for 12 h, and the survival rates of larvae were recorded. (c) Paraquat-induced nuclear translocation of TcFOXO. Beetles were treated with paraquat for 8 h and the midguts were dissected and stained with an anti-TcFOXO antibody (red) and DAPI (blue). Merged images showed the subcellular localization of TcFOXO. The protein levels of TcFOXO in nuclear and cytoplasmic proteins were detected with an anti-TcFOXO antibody and normalized with an anti-α-tubulin or anti-Histone H3 antibody. Representative blots from three independent experiments were shown. (d) Total protein and phosphoprotein stains of immunoprecipitated TcFOXO from paraquat-treatment and control samples. The gels were stained by coomassie blue (left panel) and Pro-Q Diamond (right panel). Arrows point to bands identified by lane labels. (e) Quantification of phosphorylation of immunoprecipitated TcFOXO from paraquat-treatment and control samples using phosphoprotein phosphate estimation kit. Numbers of moles of phosphorus per mole of TcFOXO were analyzed to detect the phosphorylation level of TcFOXO after treatment with paraquat. Data were expressed as mean ± SEM (n = 3 biologically independent replicates). Asterisks indicate differences statistically significant at * *P* < 0.05 and ** *P* < 0.01 (student’s t-test).

To test the involvement of FOXO in the AMPK-mediated upregulation of *AK* expression, we first investigated changes in *TcFOXO* expression in response to oxidative stress. We found that paraquat treatment led to markedly increased abundances in both mRNA and protein levels of TcFOXO (Fig 3A). We then performed RNAi to knockdown *TcFOXO* expression and measured the survivorship of the beetles after exposure to paraquat. The results showed that the knockdown of *TcFOXO* did not lead to the death of the *T*. *castaneum* under normal conditions. However, after the paraquat treatment, the survival rate of the dsFOXO-treated group was only 10.0%, which was significantly lower than the WT (56.7%) and dsEGFP (65.2%) groups (Fig 3B), demonstrating that *TcFOXO* is required for oxidative stress tolerance.

We next performed immunofluorescence experiments using midguts of female adults 8 hours after paraquat treatment to test whether paraquat-induced oxidative stress promotes nuclear translocation of TcFOXO. We found that TcFOXO was located almost exclusively in the cytoplasm of untreated beetles, whereas paraquat treatment promoted nuclear localization of TcFOXO (Fig 3C). Further western blot experiments with different subcellular fractions confirmed the result (Fig 3C). Thus, increased expression and nuclearization of TcFOXO induced by the paraquat treatment was confirmed in independent experiments.

We tested whether the paraquat-induced nuclearization of TcFOXO is associated with phosphorylation of the protein. TcFOXO was successfully purified by immunoprecipitation for the detection of the phosphorylation in the Pro-Q gel-staining [33,34]. The phosphoprotein staining revealed increased phosphorylation of TcFOXO after paraquat treatment (Fig 3D). The level of paraquat-induced phosphoprotein of TcFOXO was further quantified using a phosphoprotein phosphate estimation kit [35]. As shown in Fig 3E, compared with the control group, paraquat treatment increased the phosphorylation levels of TcFOXO protein by 3.7-fold. These data suggested that the phosphorylation-induced nuclear translocation of TcFOXO is a response to paraquat treatment.

### FOXO directly activates *AK1* and *AK2* transcription

**Fig 4:**
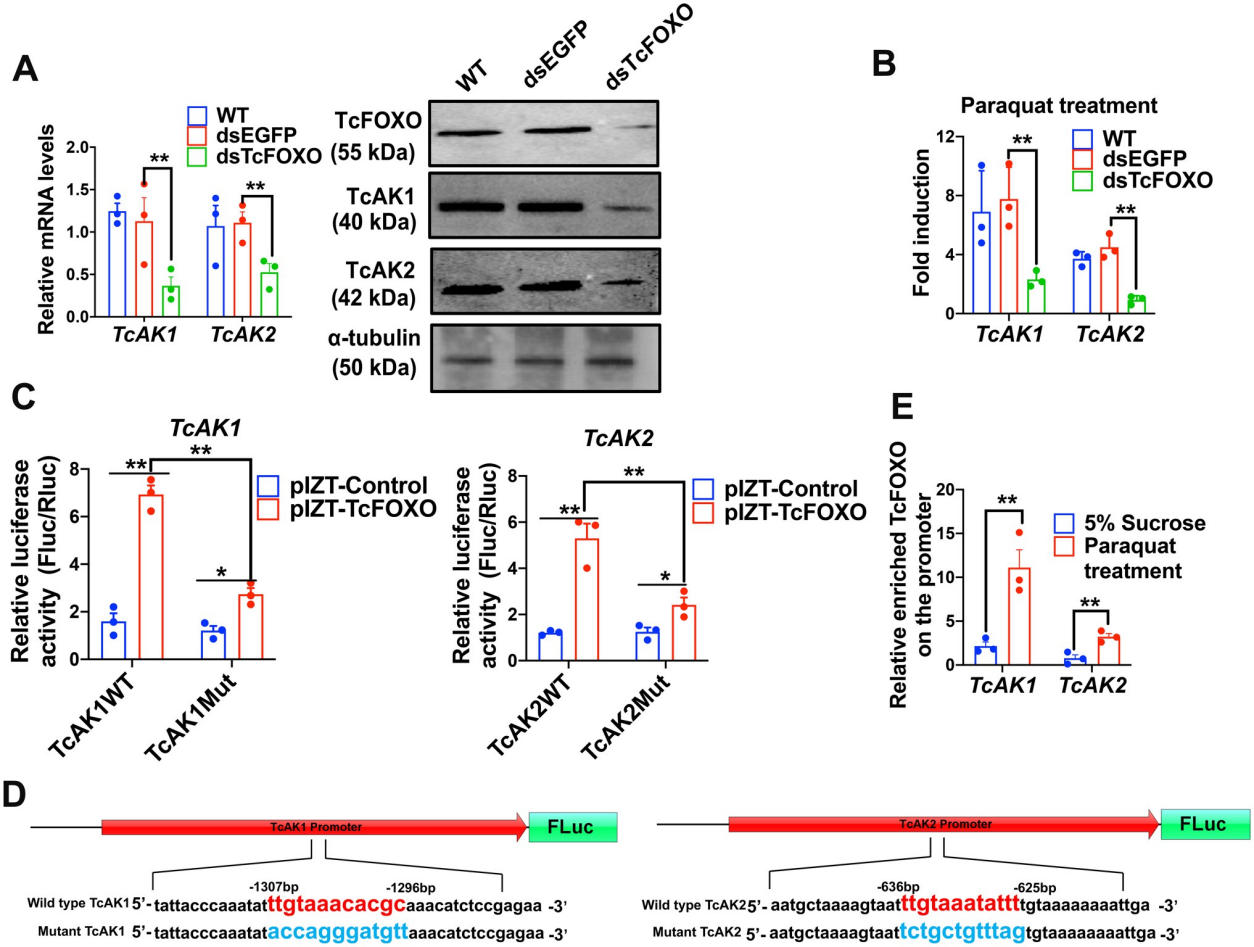
TcFOXO regulates the paraquat-induced expression of *TcAK*s. (a) Knockdown of *TcFOXO* reduced the basal mRNA and protein expression of *TcAK1* and *TcAK2*. The relative mRNA levels of *TcAK1* and *TcAK2* were determined by RT-qPCR using ribosomal protein S3 (rps3) as an internal reference. The relative protein levels of TcAK1 and TcAK2 were determined by Western blot using the anti-α-tubulin tubulin antibody as a loading control. (b) The knockdown of *TcFOXO* attenuated the paraquat-induced mRNA expression of *TcAK1* and *TcAK2*. (c) Over-expression of TcFOXO increased the WT promoter activities of *TcAK1* and *TcAK2* in the Sf9 cells and the mutation in the promoters (Mut) attenuate the induction. Sf9 cells were transfected with luciferase vectors carrying the wild-type or mutant promoter regions of TcAKs as well as the overexpression vector of TcFOXO or a control vector. Cells were harvested for the dual-luciferase reporter assay at 24 h after transfection. The relative firefly luciferase activities were measured and normalized to *Renilla* luciferase from the same cell lysates. (d) Diagram of the wild-type (WT) and mutant (Mut) *TcAK1* and *TcAK2* promoters cloned into dual luciferase reporter vectors. Predicted FOXO binding element (FBE) is indicated in red, and randomly mutated FBE is indicated in blue. (e) ChIP-qPCR analysis revealed increased bindings of TcFOXO to the *TcAK1* and *TcAK2* promoter regions after exposure to paraquat. The beetles were treated with paraquat or 5% sucrose for 8 h. TcFOXO-DNA complexes were cross-linked by formaldehyde and immunoprecipitated with the anti-TcFOXO antibody. The amounts of *TcAK1* and *TcAK2* promoter regions binding with TcFOXO was determined by qPCR and normalized with input DNA. Data were expressed as mean ± SEM (n = 3 biologically independent replicates). Asterisks indicate differences statistically significant at * *P* < 0.05 and ** *P* < 0.01 (student’s t-test).

To test the role of FOXO in the transcriptional regulation of *AK*s, we first suppressed *TcFOXO* by an injection of dsTcFOXO into 15-day-old larvae. The levels of *TcAK*s transcripts were measured under basal and oxidative stress-induced conditions. As shown in Fig 4A, knockdown of *TcFOXO* significantly decreased the basal mRNA and protein expression of TcAK1 and TcAK2 and prevented full induction of *TcAK1* and *TcAK2* after exposure to paraquat (Fig 4B).

To examine whether the TcFOXO directly regulates the transcription of *TcAKs*, we explored transcriptional factor binding elements in the promoter regions of *TcAKs* using two prediction programs, JASPAR (http://jaspar.genereg.net/) and TFBIND (http://tfbind.hgc.jp/), which led to the uncovering of several consensus FOXO binding elements (FBEs) (S1 Table). The 5′-flanking regions of *TcAK1* (from −2170 to +130 bp) and *TcAK2* (from −2079 to +88 bp) containing the FBEs were tested by using luciferase reporter in Sf9 cells for the TcFOXO-mediated upregulation. The dual-luciferase assay revealed that TcFOXO enhanced *TcAK1* and *TcAK2* promoter-driven relative luciferase expression by 4.4-fold and 4.1-fold, respectively (Fig 4C), compared to the control having empty plasmid without TcFOXO overexpression. Further random mutation of FBE sequences with the highest score value in *TcAK1* and *TcAK2* promoter regions (Fig 4D) profoundly attenuated the luciferase reporter activity induced by TcFOXO overexpression (Fig 4C).

We next performed chromatin immunoprecipitation (ChIP)-qPCR experiments to examine whether TcFOXO directly binds to the *TcAK1* and *TcAK2* promoter regions *in vivo*. The primers for ChIP-qPCR experiments were designed for targeting FBE sequences of the *TcFOXO* tested above (S2 Table). ChIP-qPCR analysis using the anti-TcFOXO antibody revealed the physical interaction between TcFOXO and the promoter regions of the *TcAK1* and *TcAK2* (Fig 4E). Remarkably, the binding of TcFOXO to the *TcAK1* and *TcAK2* promoter regions significantly increased by 5.2-fold and 4.2-fold, respectively, after paraquat treatment (Fig 4E). Therefore, these results strongly suggest that the TcFOXO is the direct activator of the *TcAK*s promoters in the oxidative stress-induced expression of *TcAK*s.

### AMPK directly phosphorylates FOXO, leading to *AKs* upregulation under oxidative stress

**Fig 5:**
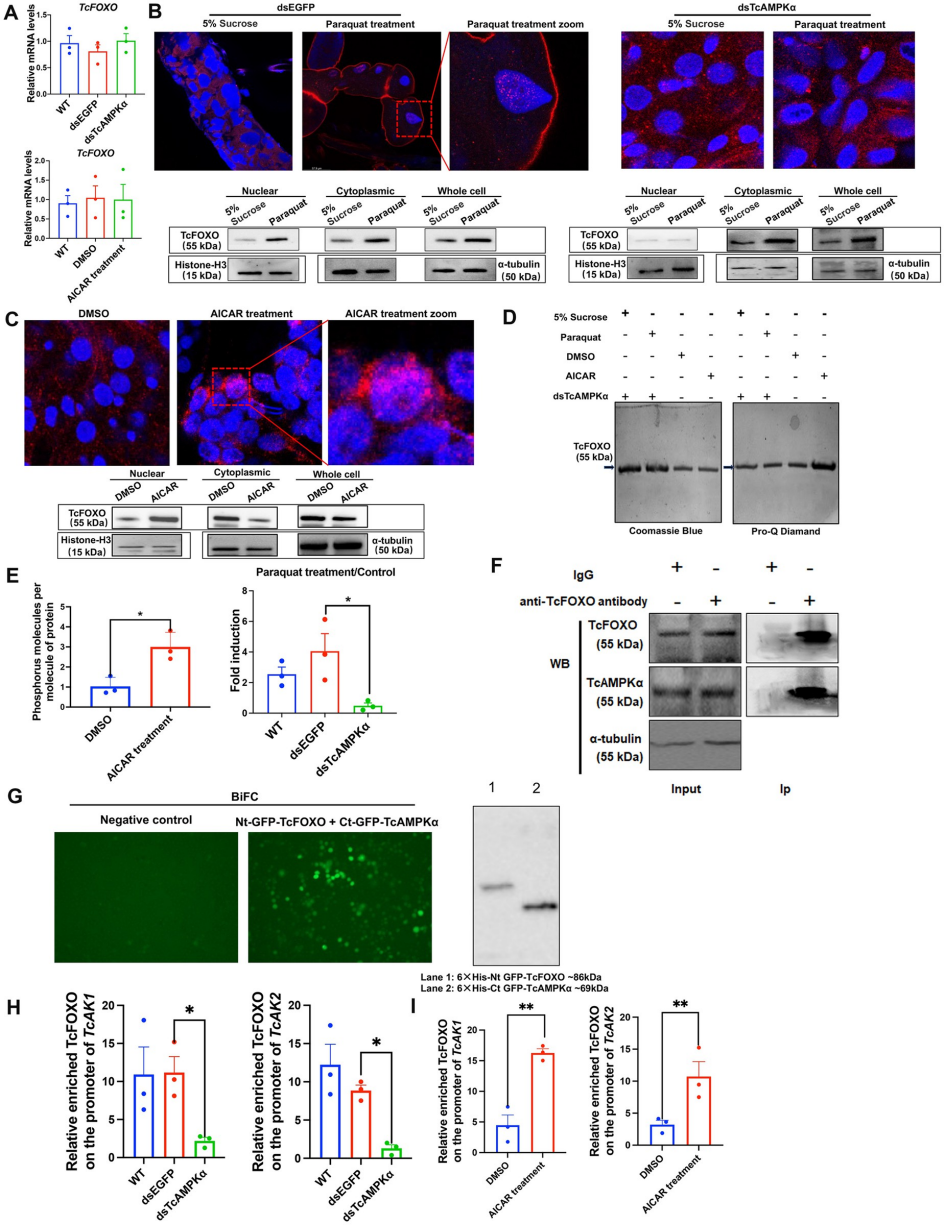
Activation of TcAMPK promotes nuclear translocation of TcFOXO by phosphorylation and mediates paraquat-induced TcFOXO recruitment to *TcAK1* and *TcAK2* promoter regions under oxidative stress conditions. (a) The relative mRNA expression of *TcFOXO* after treatment with dsTcAMPKα and AICAR. (b) Knockdown of *TcAMPKα* mRNA expression attenuated the paraquat-induced nuclear translocation of TcFOXO. (c) AICAR treatment induced the nuclear translocation of TcFOXO in beetles. Midguts from the treated and control groups were dissected and stained with an anti-TcFOXO antibody (red) and DAPI (blue). Merged images showed the subcellular localization of TcFOXO. Representative images from three independent experiments were shown. The protein level of TcFOXO in nuclear and cytoplasmic proteins was examined with an anti-TcFOXO antibody and normalized with the anti-α-tubulin or anti-Histone H3 antibody. Representative blots from three independent experiments were shown. (d) Total protein and phosphoprotein stains of immunoprecipitated TcFOXO from dsTcAMPKα-treatment, dsTcAMPKα and paraquat-treatment as well as AICAR-treatment and control samples. The same gel was stained by Pro-Q Diamond (left panel) and coomassie blue (right panel). Arrows point to bands identified by lane labels. (e) Quantification of phosphorylation of immunoprecipitated TcFOXO from AICAR-treatment as well as dsTcAMPKα and paraquat-treatment and corresponding control samples using phosphoprotein phosphate estimation kit. Numbers of moles of phosphorus per mole of TcFOXO were analyzed to detect the phosphorylation level of TcFOXO after each treatment. (f) Co-IP assay for TcAMPK–TcFOXO interaction. Co-IP assays were performed using the anti-TcFOXO antibody, then the immune complex with agarose was subjected to western blot analysis to detect the presence of TcAMPKα protein with the anti-TcAMPKα antibody. The whole-cell lysates (input) were also tested with either anti-TcFOXO (WB: TcFOXO) or anti-TcAMPKα (WB: TcAMPKα) antibodies to verify the protein expression, and α-tubulin was used as a loading control. For control experiments, the cell lysates were precipitated with nonimmune rabbit IgG and subsequently analyzed by western blot. (g) The BiFC assay for TcAMPK–TcFOXO interaction. Nt-GFP -TcFOXO and Ct-GFP-TcAMPKα plasmids were transfected into Sf9 cells. Cells were fixed and scanned at 48 h post-transfection. Western blot with anti-6×His antibody was conducted to confirm the protein expression of TcFOXO and TcAMPKα. (h) Knockdown of *TcAMPKα* mRNA expression attenuated the paraquat-induced recruitment of TcFOXO to the *TcAK1* and *TcAK2* promoter regions. (i) AICAR treatment enhanced the paraquat-induced recruitment of TcFOXO to the *TcAK1* and *TcAK2* promoter regions. Data were expressed as mean ± SEM (n = 3 biologically independent replicates). Asterisks indicate differences statistically significant at * *P* < 0.05 and ** *P* < 0.01 (student’s t-test).

The paraquat-induced phosphorylation and nuclearization of TcFOXO, together with our previous finding that paraquat treatment led to significantly increased phospho-AMPKα (p-AMPKα) levels [26], prompted us to investigate the interaction between TcAMPK and TcFOXO. We first tested whether TcAMPK affects the transcription of *TcFOXO*. We found that neither knockdown of *TcAMPKα* mRNA expression nor AICAR treatment affected the mRNA expression of *TcFOXO* (Fig 5A), implying no transcriptional activation of *TcFOXO* by TcAMPK. We then tested whether TcAMPK affects TcFOXO subcellular localization under oxidative stress condition. Immunofluorescence for TcFOXO showed that the knockdown of *TcAMPKα* mRNA expression significantly inhibited the nuclear translocation of TcFOXO induced by paraquat (Fig 5B). The western blot experiments also showed that in the dsTcAMPKα injection group, the abundance of TcFOXO increased significantly in the cytoplasm rather than in the nucleus after paraquat treatment (Fig 5B), implying the presence of TcAMPK-independent upregulation of TcFOXO by paraquat, but limited in the cytoplasm. We next tested the effects of AICAR treatment on subcellular localization of TcFOXO and found that AICAR treatment significantly promoted the translocation of TcFOXO from the cytoplasm to the nucleus (Fig 5C). Western blot experiments with different cell fractions also detected an increase in the TcFOXO abundance in the nucleus whereas its total protein content remained the same after AICAR treatment (Fig 5C).

We further tested whether TcAMPK affects TcFOXO subcellular localization via phosphorylation of the TcFOXO. We first detected the phosphorylation level of immunoprecipitated TcFOXO with the Pro-Q gel-staining method. We found that AICAR treatment significantly increased TcFOXO phosphorylation level, whereas knockdown of *TcAMPKα* mRNA expression significantly attenuated the paraquat-induced TcFOXO phosphorylation level (Fig 5D). This result was further verified by quantification of phosphorylation of immunoprecipitated TcFOXO. We found that AICAR treatment resulted in a 3.0-fold increase in TcFOXO phosphorylation level compared to the control, whereas knockdown of *TcAMPKα* mRNA expression significantly reduced the paraquat-induced TcFOXO phosphorylation level by 87.5% (Fig 5E). We then tested whether TcAMPK directly interacted with TcFOXO by using co-immunoprecipitation (Co-IP) assay. As shown in Fig 5F, endogenous TcAMPKα was co-immunoprecipitated with TcFOXO from the whole insect homogenates, suggesting the direct interaction *in vivo*. Additionally, paraquat treatment enhanced the interaction between TcAMPKα and TcFOXO to some extent (S2 Fig). This result was further verified by the bimolecular fluorescence complementation (BiFC) assay. The GFP fluorescence signal was detected only when the C-terminal half of GFP fused with TcAMPKα was co-expressed with the N-terminal half of GFP fused with TcFOXO (Fig 5G). In the negative control, cell line co-transfected with the C-terminal half of GFP and the N-terminal half of GFP did not show fluorescence (Fig 5G). Additionally, AMPK-dependent phosphorylation sites have been identified in *Caenorhabditis elegans* DAF-16 (Thr 166, Ser 202, and Ser 314) [31] and *Homo sapiens* FOXO3 (Thr 179 and Ser 215) [36]. Multiple sequence alignments revealed that these phosphorylation sites are conserved in TcFOXO (S3 Fig), providing further evidence that TcFOXO is the putative substrate of TcAMPKα.

Finally, combining the AMPK-FOXO-AK gene regulatory cascades, we tested whether AMPK activity regulates FOXO binding on the *AKs* promoter regions. We found that the knockdown of *TcAMPKα* significantly reduced the degree of paraquat-induced TcFOXO enrichment to the *TcAK1* and *TcAK2* promoter regions by 80.4% and 85.3%, respectively (Fig 5H). In contrast, AICAR treatment significantly increased the enrichment of TcFOXO to the *TcAK1* and *TcAK2* promoter regions by 3.6-fold and 3.3-fold, respectively (Fig 5I). Taken together, TcAMPK enhances TcFOXO recruitment to the *TcAK1* and *TcAK2* promoter regions primarily by direct phosphorylation of TcFOXO to promote its nuclear translocation under oxidative stress conditions.

### AMPK directly interacts with AKs

**Fig 6:**
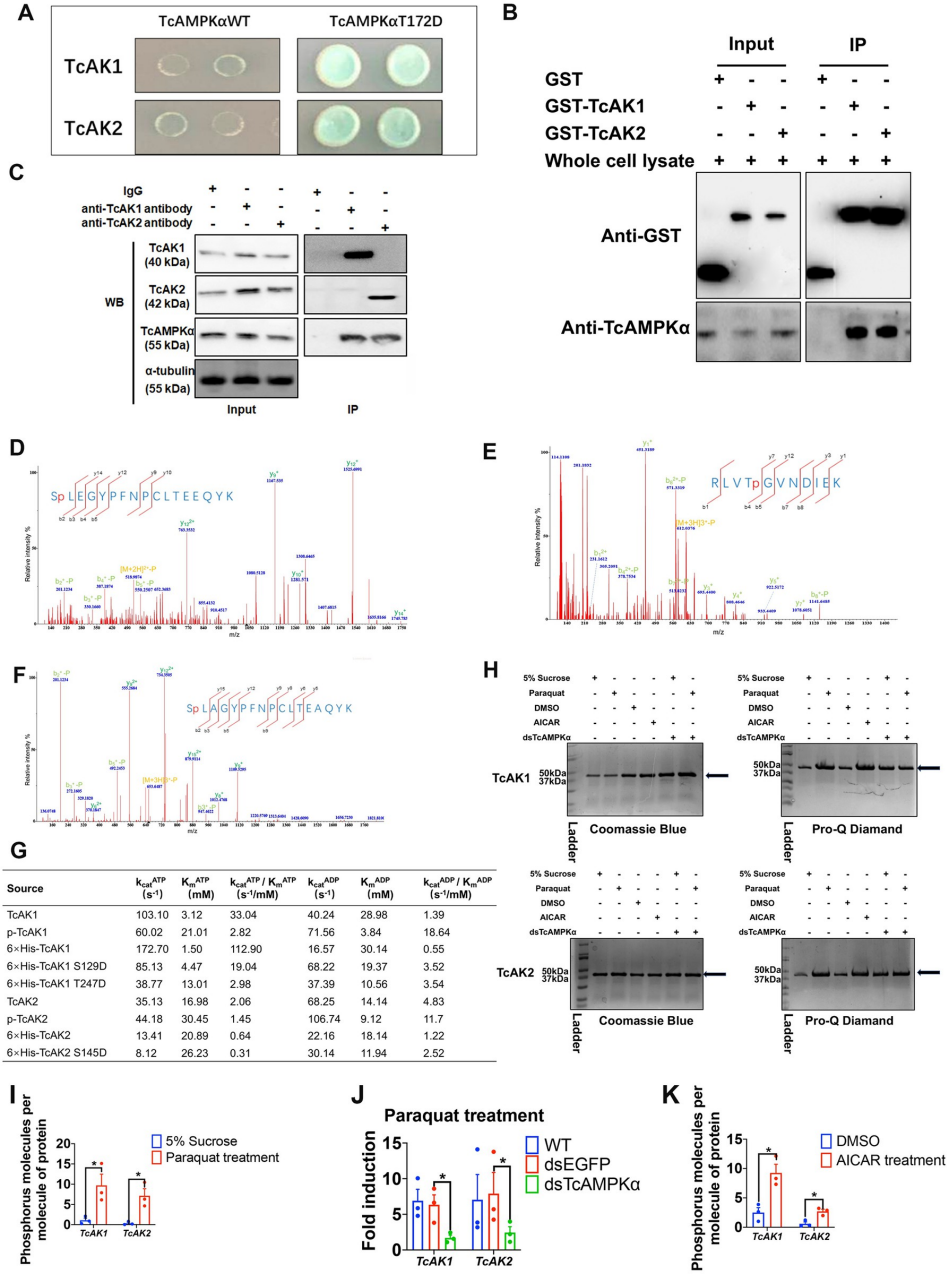
TcAMPK interacts directly with TcAKs and alters their enzymatic activity through phosphorylation. (a) Yeast-two-hybrid assay for TcAMPK–TcAKs interaction. The wild-type (WT) and constitutively active mutant (T172D) TcAMPKα were used to analyze the interactions with TcAK1 and TcAK2. Yeasts were grown on media for 72 h at 30°C. The grown spots represent the interactions of TcAMPKαT172D with TcAKs, while no interaction was found between TcAMPKα (WT) and TcAK1 or TcAK2. Yeast cells were grown on SD-WL media lacking tryptophan and leucine and SD-AHWL media lacking adenine, histidine, tryptophan, and leucine to allow verification of the incorporation of bait and prey plasmids (growth) and bait/prey interaction (selection). (b) GST-pull down assay for TcAMPK–TcAKs interaction. Whole lysates from *T*. *castaneum* cells were incubated with the GST-TcAK1 or GST-TcAK2 fusion proteins as indicated as well as GST alone (negative control) and were analyzed by western blot with an anti-AMPK α antibody (lower panel) and an anti-GST antibody as protein controls (upper panel), respectively. (c) Co-IP assay for TcAMPK–TcAKs interaction. Co-IP assays were performed using an anti-TcAK1 or TcAK2 antibody, then the immune complex with agarose was subjected to western blot analysis to detect the presence of TcAMPKα protein with the anti-TcAMPKα antibody. The whole-cell lysates (input) were also tested with anti-TcAK1 (WB: TcAK1), anti-TcAK2 (WB: TcAK2), or anti-TcAMPKα (WB: TcAMPKα) antibody to verify the protein expression, and α-tubulin was used as a loading control. For control experiments, the cell lysates were precipitated with nonimmune rabbit IgG and subsequently analyzed by western blot. (d, e, f) *In vitro* AMPK-dependent phosphorylation of TcAK1 at S129 and T247 as well as TcAK2 at S145. The phosphorylated TcAK1 and TcAK2 were excised by trypsin and analyzed by MALDI-TOF/TOF-MS/MS or LC-MS/MS. MS/MS spectrum showed fragmentation of SLEGYPFNPCLTEEQY (m/z = 518.9872, z = +2, RT = 100.77) and RLVTGVNDIEK (m/z = 644.6982, z = +3, RT = 30.76) in TcAK1 and SLAGYPFNPCLTEAQYK (m/z = 435.7658, z = +3, RT = 103.47) in TcAK2. (g) The enzymatic constants of *in vitro* expressed wild-type proteins (6×His-TcAK1 and 6×His-TcAK2) and mutant proteins (6×His-TcAK1 S129D, 6×His-TcAK1 T247D, and 6×His-TcAK2 S145D), as well as TcAK1 and TcAK2, purified from AICAR-treated groups (p-TcAK1 and p-TcAK2) and the control groups (TcAK1 and TcAK2). (h) The detection of the effect of paraquat, AICAR treatment on TcAK1 and TcAK2 phosphorylation levels, and the effect of *TcAMPKα* knockdown on induced-TcAK1 or TcAK2 phosphorylation levels by paraquat treatment using Pro-Q Diamond phosphoprotein-stained kit. The gels were shown stained by coomassie blue (left panel) and Pro-Q Diamond (right panel). Arrows point to bands identified by lane labels. (i) Compared to the control group, paraquat treatment significantly increased the phosphorylation levels of TcAK1 and TcAK2, which were detected by phosphoprotein phosphate estimation kit. (j) Knockdown of *TcAMPKα* mRNA expression significantly attenuated the paraquat-induced up-regulation of the phosphorylation levels of TcAK1 and TcAK2. (k) AICAR treatment significantly up-regulated the phosphorylation levels of TcAK1 and TcAK2 compared to the control group. Data are expressed as mean ± SEM (n = 3 biologically independent replicates). Asterisk indicates differences statistically significant at *P< 0.05 (student’s t-test).

Considering that AMPK can interact directly with CK, the mammalian orthologue of AK [36], we further investigated the possibility of the direct action of AMPK on AK as a parallel pathway of the transcriptional regulation described above. A yeast two-hybrid assay was performed to test interactions between TcAMPK and TcAKs. The assays revealed that both AK1 and AK2 can interact with constitutively active TcAMPKα T172D, which is a mutant AMPK mimicking phosphorylated AMPK [32,37], whereas wild-type TcAMPKα did not show the interaction (Fig 6A). In addition, in a GST pull-down assay to test the *in vivo* interactions, respective GST-TcAK1 and GST-TcAK2 fusion proteins pulled down native TcAMPKα from the whole cell lysate protein of the larvae (Fig 6B). Moreover, Co-IP also verified the physical interaction between TcAMPKα and TcAKs (Fig 6C), and paraquat treatment enhanced this interaction (S4 Fig). Collectively, these results indicate the direct interaction of TcAMPKα with TcAK1 and TcAK2.

### AMPK directly phosphorylates AKs and alters the enzymatic activity

Direct molecular interactions between AMPK and AKs led to the test of the possibility of AMPK-mediated phosphorylation of AKs. We performed *in vitro* phosphorylation assay in incubation of the purified constitutively active His-tagged TcAMPKαT172Dβγ with TcAK1 or TcAK2. The phosphorylation patterns of immunoprecipitated TcAK1 and TcAK2 were analyzed by matrix-assisted laser desorption ionization-time of flight tandem mass spectrometry (MALDI-TOF MS/MS) or liquid chromatography with tandem mass spectrometry (LC-MS/MS). This approach revealed three phosphopeptides including 129-SpLEGYPFNPCLTEEQYK-145 and 244-RLVTpGVNDIEK-254 in TcAK1 as well as 145-SpLAGYPFNPCLTEAQYK-161 in TcAK2 (Fig 6D, 6E and 6F). Further analysis revealed that the residue corresponding to the common phosphorylated site in TcAK1 (S129) and TcAK2 (S145) is highly conserved not only in all other insect Aks but also in PKs of all other species that have been analyzed, including *C*. *elegans* AK and *H*. *sapiens* brain-type CK (CKB) and muscle-type CK (CKM). The residue corresponding to the second phosphorylated site in TcAK1 (T247) is also conserved in *H*. *sapiens* CKB, but not found in TcAK2 and CKM as well as several other species analyzed (S1 Fig).

To address the functional relevance of the identified phosphosite in TcAK1 and TcAK2, the Ser129 and T247 in TcAK1 and Ser145 in TcAK2 were replaced by phosphor-mimetic aspartic acid (TcAK1 S129D, T247D, and TcAK2 S145D) to generate the mock-phosphorylated proteins. We tested the influence of the phosphosite on kinetic constants of TcAK1 and TcAK2 *in vitro*. Our results showed that the K_m_ values of WT, mutant TcAK1 S129D, and mutant TcAK1 T247D were 1.5 mM, 4.5 mM, and 13.0 mM for ATP, 30.1 mM, 19.4 mM, and 10.6 mM for ADP, respectively (Fig 6G), resulting in the changes of ATP: ADP ratio of the K_m_ from 0.05 in non-phospho TcAK1 to 0.2 in mutant TcAK1 S129D and 1.2 in mutant TcAK1 T247D (Fig 6G). The K_m_ values of WT and mutant TcAK2 S145D were 20.9 mM and 26.2 mM for ATP, 18.1 mM and 11.9 mM for ADP, respectively (Fig 6G), resulting in the changes of ATP: ADP ratio of the K_m_ from 1.2 in non-phospho TcAK2 to 2.2 in TcAK2 S145D.

We next investigated the role of TcAMPK in TcAKs phosphorylation under oxidative stress conditions *in vivo*. The phosphoprotein staining using Pro-Q Diamond phosphoprotein gel stain kit revealed that paraquat treatment markedly increased the phosphorylation levels of TcAK1 and TcAK2, whereas knockdown of *TcAMPKα* mRNA expression markedly suppressed the paraquat-induced TcAK1 and TcAK2 phosphorylation (Fig 6H). We then treated insects with AICAR and found significantly increased phosphorylation levels of TcAK1 and TcAK2 relative to those in DMSO-treated controls (Fig 6H). The changes in phosphorylation levels were further examined using a phosphoprotein phosphate estimation kit. Paraquat treatment increased the phosphorylation levels of TcAK1 and TcAK2 by 8.8-fold and 23.7-fold, respectively, compared to the control group (Fig 6I), whereas knockdown of *TcAMPKα* mRNA expression significantly suppressed the paraquat-induced TcAK1 and TcAK2 phosphorylation by 73.3% and 69.1%, respectively (Fig 6J). We also found that AICAR treatment significantly enhanced the phosphorylation levels of TcAK1 and TcAK2 (3.7 and 4.5 folds, respectively, Fig 6K). We further tested the effects of AICAR treatment on TcAK1 and TcAK2 enzyme activity. We extracted proteins from the AICAR-treated and the control group insects and then purified TcAK1 and TcAK2 proteins by immunoprecipitation using anti-TcAK1 antibody or anti-TcAK2 antibody, respectively. Compared with the DMSO treatment group, AICAR treatment resulted in an increase in K_m_ value of ATP from 3.1 mM to 21.0 mM and a decrease in K_m_ value of ADP from 29.0 mM to 3.8 mM for TcAK1 (Fig 6G). A similar result was also observed for TcAK2. The K_m_ value of ATP increased from 17.0 mM to 30.5 mM, whereas the K_m_ value of ADP decreased from 14.1 mM to 9.1 mM (Fig 6G). Thereby, the changes in the ATP: ADP ratio of the K_m_ were from 0.1 in the control group to 5.5 in the AICAR-treated group for TcAK1 and from 1.2 to 3.4 for TcAK2, respectively.

**Fig 7:**
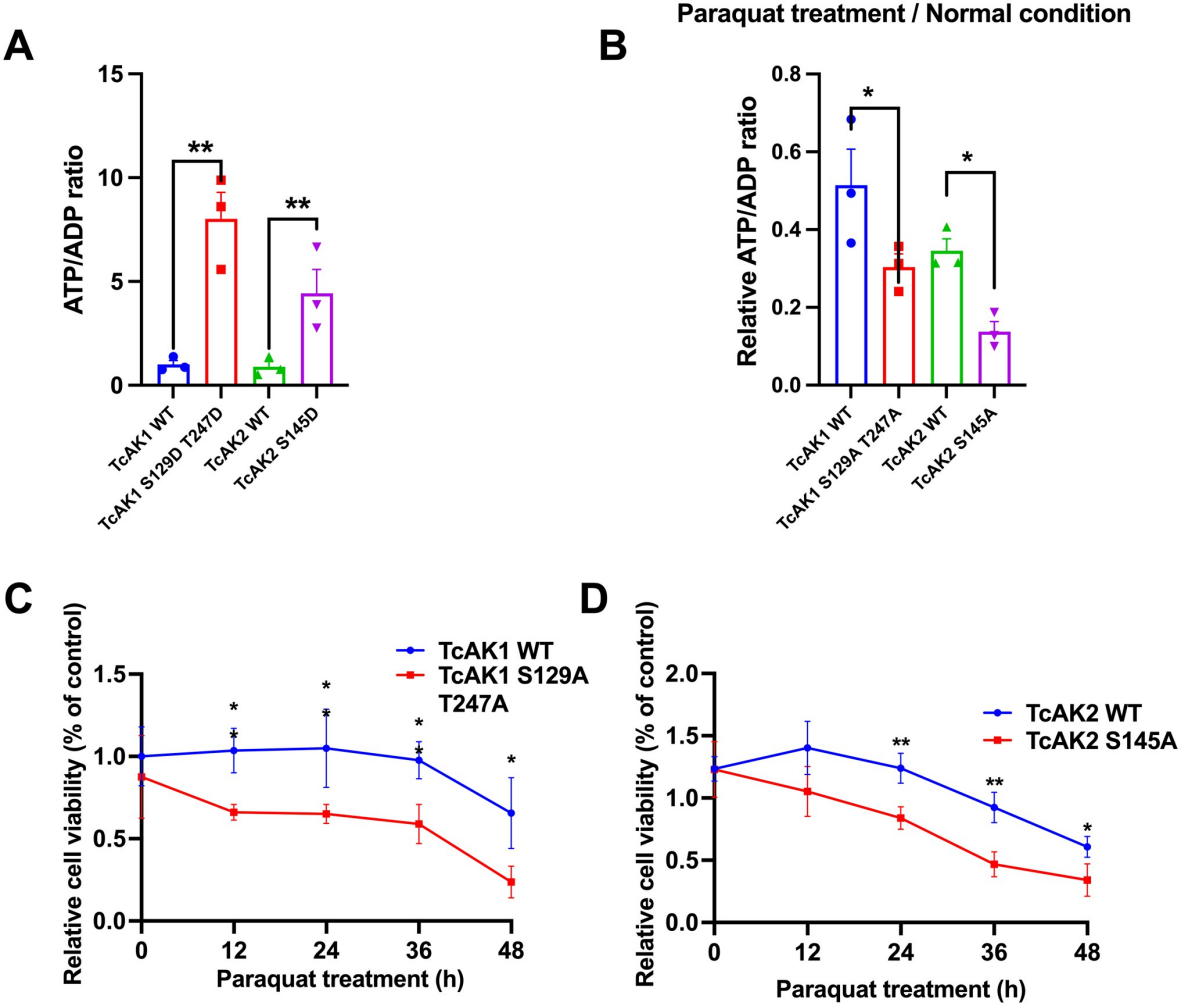
AMPK-dependent phosphorylation of TcAKs increases the ATP/ADP ratio and improves the cell viability under oxidative stress conditions. (a) The effects of transfection of phospho-mimetic TcAK1 or TcAK2 mutants into the Sf9 cells on the ATP/ADP ratio under normal conditions. (b) The effects of transfection with phospho-deficient TcAK1 or TcAK2 mutant on the ATP/ADP ratio of Sf9 cells after H_2_O_2_ treatment. (c) The cell viability of the Sf9 cells transfected with phospho-deficient TcAK1 mutant in the presence of H_2_O_2_. (d) The cell viability of the Sf9 cells transfected with phospho-deficient TcAK2 mutant in the presence of H_2_O_2_. Data are expressed as mean ± SEM (n = 3 biologically independent replicates). Asterisk indicates differences statistically significant at **P< 0.01 and *P< 0.05 (student’s t-test).

We further tested the effects of the overexpression of the phosphor-mimetic or phosphor-deficient TcAK mutants on the ATP and ADP content as well as cell survival in Sf9 cells. The results showed that overexpression of phosphor-mimetic proteins TcAK1 S129D T247D and TcAK2 S145D upregulated the ATP/ADP ratio by 7.02-fold and 3.97-fold, respectively (Fig 7A). Upon exposure to paraquat in Sf9 cells transfected with vectors expressing phosphor-deficient proteins or WT proteins, overexpression of TcAK1 S129A T247A and TcAK2 S145A led to a 75.75% and 59.34% decrease in the ATP/ADP ratio, respectively, compared to the control group (Fig 7B). Additionally, the proliferation rate of Sf9 cells exposed to paraquat was evaluated by Cell Counting Kit-8 (CCK-8) assay. The results showed that the cell viability of Sf9 cells transfected with TcAK1 S129A T247A was significantly lower than the control group after 12 h, 24 h, 36 h, and 48 h of paraquat treatment, which was 66.10%, 65.09%, 58.96%, and 23.73% of the control group (Fig 7C), respectively. We also found that the cell viability of cells transfected with TcAK2 S145A was only 68.11%, 37.84%, and 27.57% of the control group after 24 h, 36 h, and 48 h of paraquat treatment, respectively (Fig 7D).

These results indicate that the phosphorylation of TcAK1 and TcAK2 by TcAMPK activation results in switches of the substrate affinities and consequently the enzyme activities. Phosphorylation converted the AK-catalyzed forward reaction, ATP to ADP, by non-phospho-TcAK1 and -TcAK2, to the reverse reaction, ADP to ATP, by the phosphor-TcAK1 and-TcAK2 to respond to energy dysregulation due to oxidative stress.

### AMPK-FOXO-CKB pathway is involved in the maintenance of ATP homeostasis in HEK293 cells

**Fig 8:**
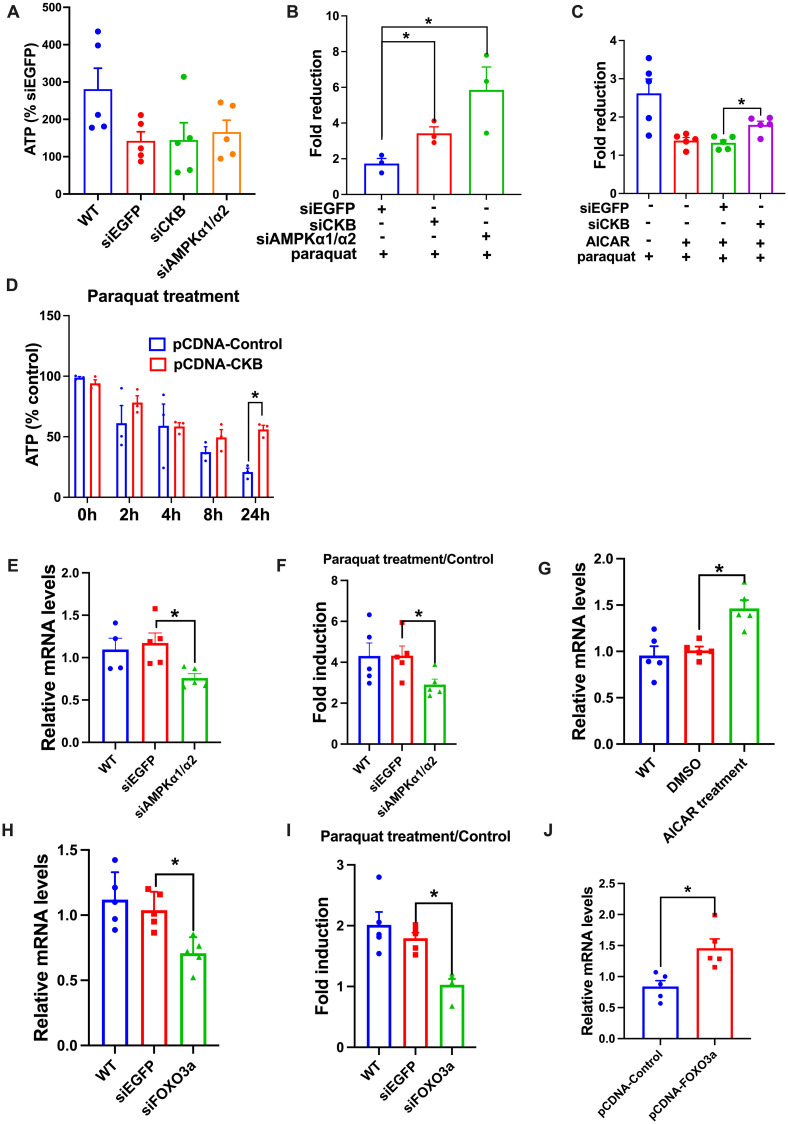
ATP homeostasis is maintained by AMPK-FOXO-CKB pathway in HEK293 cells under oxidative stress conditions. **(a)** Knockdown of *CKB* or *AMPKα1*/*α2* mRNA expression did not affect the basal ATP content in HEK293 cells. **(b)** Knockdown of *CKB* or *AMPKα1*/*α2* mRNA expression increased the fold of ATP decrease caused by paraquat treatment for 24h in HEK293 cells. **(c)** Knockdown of *CKB* mRNA expression attenuated the AICAR-induced increase in ATP content under paraquat treatment in HEK293 cells. **(d)** Overexpression of CKB significantly rescued the reduction of ATP content caused by paraquat treatment in HEK293 cells. **(e)** Knockdown of *AMPKα1*/*α2* mRNA expression down-regulated the basal mRNA expression of *CKB* in HEK293 cells. **(f)** Knockdown of *AMPKα1*/*α2* mRNA expression attenuated the paraquat-induced mRNA expression of *CKB* in HEK293 cells. **(g)** 24 hours of AICAR treatment increased the mRNA expression of *CKB*. **(h)** Knockdown of *FOXO3a* reduced the basal mRNA expression of *CKB* in HEK293 cells. **(i)** Knockdown of *FOXO3a* attenuated the paraquat-induced mRNA expression of *CKB* in HEK293 cells. **(j)** Over-expression of FOXO3a increased the mRNA expression of *CKB* in HEK293 cells. mRNA expression was determined by qPCR using GAPDH as an internal reference. Data were expressed as mean ± SEM (n = 3 biologically independent replicates). Asterisks indicate differences statistically significant at * *P* < 0.05 (student’s t-test).

We further tested the role of AMPK and CK, the mammalian homolog of AK, in the maintenance of ATP homeostasis under oxidative stress conditions in HEK293 cells. Consistent with our findings in *T*. *castaneum*, knockdown of CKB or AMPKα1/α2 did not affect basal ATP content (Fig 8A), whereas significantly increased ATP depletion induced by paraquat or H_2_O_2_ (Figs 8B and S5A). We also found that knockdown of *CKB* markedly attenuated the AICAR-induced stability of ATP content, whereas overexpression of CKB in HEK293 cells led to stabilization of ATP content under oxidative stress conditions induced by paraquat or H_2_O_2_ (Figs 8C, 8D, S5B and S5C).

We next tested the role of AMPK and FOXO in the transcriptional regulation of *CKB*. Slightly different from *T*. *castaneum*, knockdown of *AMPKα1*/*α2* decreased basal *CKB* expression (Fig 8E). As expected, knockdown of *AMPKα1*/*α2* attenuated paraquat- or H_2_O_2_-induced *CKB* expression (Figs 8F and S5D), and AICAR treatment significantly upregulated CKB expression (Fig 8G). Knockdown of *FOXO3a* in HEK293 cells also reduced the mRNA expression of *CKB* under either normal (Fig 8H) or oxidative stress conditions induced by paraquat or H_2_O_2_ (Figs 8I and S5E), whereas overexpression of FOXO3a in HEK293 cells significantly upregulated mRNA expression of *CKB* (Fig 8J). These results suggested that the regulatory axis of AMPK-FOXO-CKB might be evolutionarily conserved in HEK293 cells.

## Discussion

**Fig 9:**
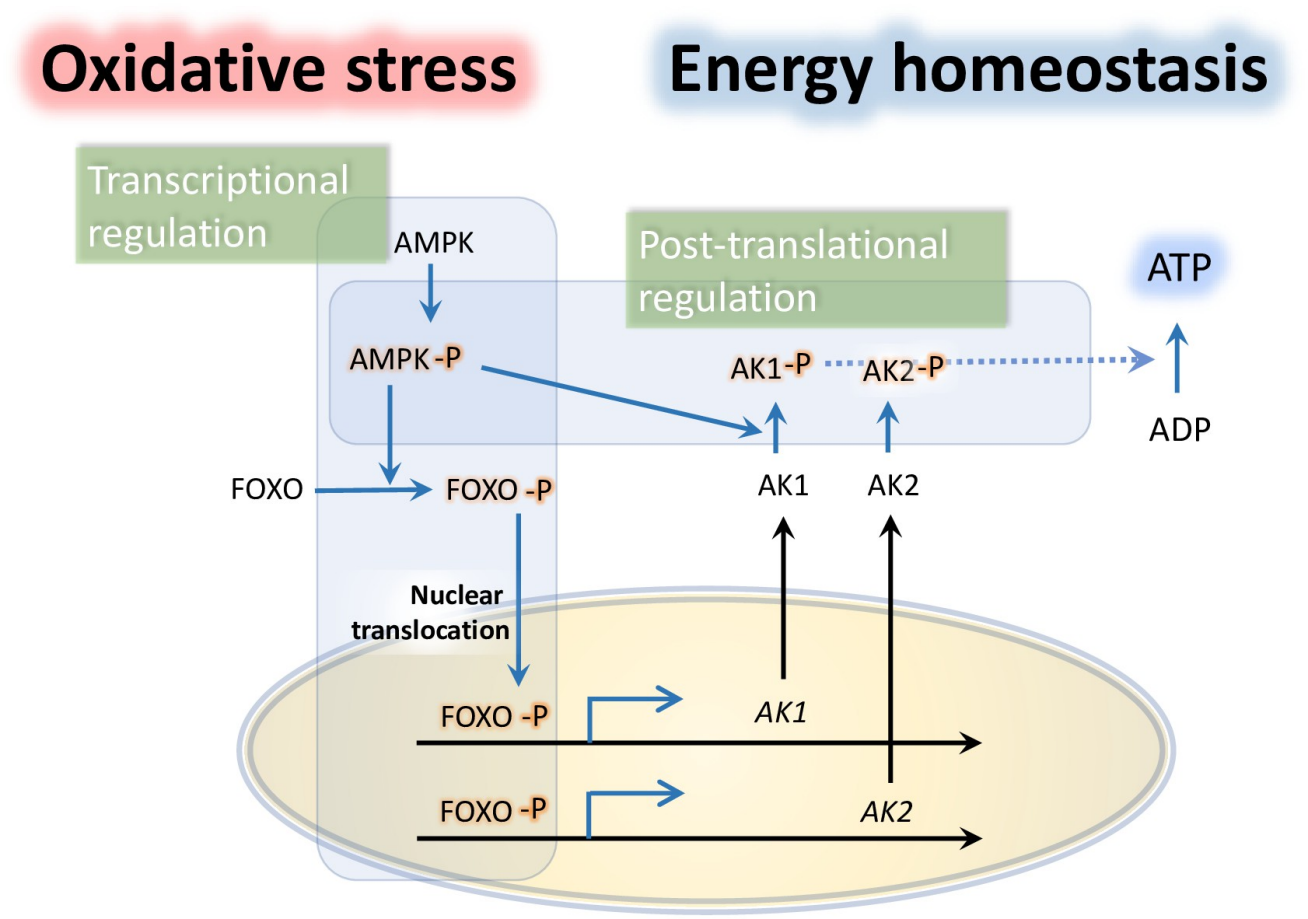
A schematic diagram illustrating the proposed AMPK/AK pathways maintaining ATP homeostasis under oxidative stress. Activation of AMPK increases the transcription level of TcAK1 and TcAK2 via phosphorylating FOXO and promoting its nuclear translocation. AMPK can also directly phosphorylate TcAK1 and TcAK2 to change their catalytic activity for rapid production of ATP.

It is well known that both AMPK and PK systems play important roles in maintaining cellular and organismal energy homeostasis [7,17,37]. In the present study, we delineated the cascades of two independent pathways from AMPK to AK synergizing the ultimate outcome of ATP homeostasis under oxidative stress condition: transcriptional regulation of *AK*s through AMPK-FOXO axis and direct phosphorylation of AKs by AMPK (Fig 9). The study using an insect model system *T*. *castaneum* successfully provided the insights into the mechanisms by taking advantage of the presence of conserved AMPK and AK system (counterpart of CK of vertebrate) with the single copy AMPK genes and robust post-embryonic RNAi technique available for suppression of the target genes in the beetle.

### Conserved FOXO function in AMPK-FOXO-PK cascade

Based on our study for the processes in the cascade actions of AMPK-FOXO-PK, oxidative stress upregulates the expressions of AMPK [26] and FOXO and activates the AMPK phosphorylation [26]. Phospho-AMPK activates phosphorylation and nuclearization of FOXO that directly binds to the promoters of AKs to increase the transcripts (Fig 9).

FOXO has been implicated in several biological processes including cell cycle, apoptosis, energy metabolism, autophagy, and insulin/insulin-like growth factor signaling [38–41]. FOXO is also involved in the cellular response to extracellular or intracellular stress stimuli including oxidative stress [42,43]. There are also cases where the immediate downstream targets of FOXO transcriptional factor include antioxidant enzymes. FOXO members respond against oxidative stress via the transcriptional regulation of manganese superoxide dismutase and catalase gene expression in human colon carcinoma cells [44]. In *Drosophila*, FOXO-induced up-regulation of neuronal expression of *Jafrac1*, a *Drosophila* homolog of human *Peroxiredoxin II*, significantly reduces the ROS level and restores mitochondrial function in response to the paraquat-induced oxidative stress [45].

In this study, we found that paraquat treatments significantly increased the mRNA and protein abundance of TcFOXO (Fig 3A), which is likely through a TcAMPK-independent pathway, and promoted phosphorylation and nuclear translocation of TcFOXO in a TcAMPK-dependent manner. The AMPK-dependent activation of FOXO transcriptional factor is likely a conserved mechanism. There have been studies, in mammals, showing AMPK-mediated phosphorylation of FOXO leading to the activation of its transcriptional activity in a rapid response to stress-induced energy deficit [38,41,43,46,47]. On the other hand, a different mechanism of FOXO is also known in the **HEK293** cells, in which AMPK regulates the expression of ferroportin 1 (*Slc40a1*) and DNA damage-induced transcript 45a (*Gadd45a*) by phosphorylating FOXO3, but without the effect on its subcellular localization [31]. Interestingly, in *C*. *elegans*, AMPK phosphorylates DAF-16, the orthologue of the human FOXO3a, at multiple sites, and DAF-16 has an effect of positive feedback loop for the expression of AMPK [48].

Despite the well-known function of the AKs in energy metabolism, the transcriptional regulation of AKs has remained largely unknown. In this study, we demonstrate that TcFOXO can directly bind to the promoter regions of *TcAK1* and *TcAK2* to regulate their transcription. We also found the expression of CKB is regulated by FOXO3a in HEK293 cells under basic and oxidative stress conditions, suggesting that this regulatory pathway is likely conserved from insects to mammals. As the biological role of FOXOs has been predominately involved in the responses to stress conditions rather than being a mediator of normal physiology [38,49], the AK upregulations as a part of the maintenance of energy homeostasis under oxidative stress seem to be an important downstream effector of FOXO. Although our study showed that AMPK-mediated FOXO activation is likely the major mechanism of AK upregulation, the FOXO activation may also happen through an alternative pathway. Notably, it has been reported in mammals that FoxO4 is phosphorylated by c-Jun-N-terminal kinases (JNK) under conditions of oxidative stress, stimulating FoxO4 nuclear accumulation [44]. Thus, whether the JNK-FOXO pathway, in addition to the AMPK-FOXO pathway, orchestrates transcriptional changes of AK to adapt to oxidative stress needs further investigation.

### Direct phosphorylation of AK by AMPK in restoration of energy homeostasis

Direct phosphorylation of AKs by AMPK, in addition to the upregulation of AK expressions discussed above, is found to accelerate the ATP generation for energy homeostasis in this study (Fig 6). In previous studies, direct interaction between AMPK and CK, the AK orthologue in mammals, has been studied [37,50]. In this study, both Y2H and GST pull-down revealed a physical interaction between TcAMPK and TcAKs, and co-immunoprecipitation of AMPK and TcAKs strongly supported the notion of their interaction in living cells. Moreover, we identified Ser129 and Thr247 in TcAK1 as well as Ser145 in TcAK2 as AMPK-dependent phosphorylation sites by using an *in vitro* kinase assay. Interestingly, the AMPK-dependent phosphorylation site shared by TcAK1 (Ser129) and TcAK2 (Ser145) corresponds to the highly conserved residue in PKs of all species analyzed including is *C*. *elegans* AK (Ser200) as well as *H*. *sapiens* CKB and CKM (Ser136). The residue corresponding to the second phosphorylation site of TcAK1 (Thr247) is also conserved in a variety of organisms including *H*. *sapiens* CKB. The conservation of these residues across phylogenetically distant organisms might indicate their *functionally important roles in enzyme activity*. In addition to the phosphorylation sites identified in this study, there are likely more numbers of phosphorylated residues in TcAK1 and TcAK2 based on the average number of phosphates detected after paraquat treatment, for which our MALDI-TOF MS/MS or LC-MS/MS analyses for phosphopeptide-enriched peptides were unable to identify. The limited number of phosphorylated sites identified in this study might be due to the relatively low activity of AMPKαT172Dβγ complexes expressed *in vitro* [31,32].

Due to increased negative charge through the phosphate, phosphorylation might induce a conformational change by creating new electrostatic interactions that greatly affects catalytic activity and substrate affinity of an enzyme [51]. It has been reported in the freshwater crayfish, *Orconectes virilis*, that anoxia-induced AK phosphorylation significantly increased K_m_ of muscle AK for L-arginine [52,53]. In the present study, we found that oxidative stress significantly increased the AMPK-dependent phosphorylation levels of TcAK1 and TcAK2, and phosphorylation of TcAK1 and TcAK2 increased K_m_ value for ATP but decreased K_m_ value for ADP, which results in a shift of the reversible reaction favoring ATP generation. Further experiments in the Sf9 cells also demonstrated that AMPK-mediated phosphorylation of TcAK1 and TcAK2 contributed to maintaining the ATP/ADP ratio and improved the cell viability under oxidative stress conditions. Both the identified phosphorylation sites in TcAK1 (Ser129 and Thr247) are located in the large C-terminal domain and in the vicinity of three (Arg123, Arg125, and Arg228) of five fully conserved arginines that form the nucleotide phosphate-binding pocket [54–56]. Interestingly, while the phosphorylation of CKB at Ser6 by AMPK did not affect its enzymatic activity [37], an *in vitro* phosphorylation assay revealed that AMPK phosphorylates and inactivates the CKM, and the residue Ser136 corresponding to the phosphorylation site in TcAK1 (Ser129) and TcAK2 (Ser145) has been identified as a potential AMPK-dependent phosphorylation site in CKB and CKM [50]. Thus, we propose that AMPK-mediated phosphorylation of PKs might react rapidly to an acute energy challenge such as oxidative stress in invertebrates and vertebrates, which is different from classical AMPK pathways involved in long-term adaptations [37].

In summary, we demonstrate that AMPK mediates transcriptional regulation of AKs via phosphorylating FOXO and promoting its nuclear translocation and its recruitment to the *AK* promoter (Fig 9). In addition, AMPK exerts direct phosphorylation of AKs for switching the enzymatic activity for ATP production (Fig 9). These results point to two separate AMPK-mediated mechanisms, AK upregulation, and activation, synergistically prevent ATP depletion under oxidative stress condition.

## Materials and methods

### Insect strain

The Georgia-1 (GA-1) strain of *T*. *castaneum* was reared on 5% (w/w) yeasted flour at 30°C and 40% relative humidity under standard conditions [57].

### Antibodies and Western blotting

For Western blotting, equal amounts of protein extracts from whole insects were separated by SDS-PAGE and transferred onto PVDF. The membranes were blocked, incubated with the corresponding primary antibody. The primary antibodies for western blots were diluted with 5% skim milk in 0.1% Tween/TBS for anti-TcAK1 (1:1,000), anti-TcAK2 (1:2,000), anti-TcFOXO (1:1,000), anti-TcAMPKα (1:1,000), anti-α-tubulin (1:5,000) (Proteintech Group, Inc., Chicago, IL, USA), anti-Histone H3 (1:5,000) (Beyotime, Shanghai, China), Mouse Anti-AMPK alpha 1 + AMPK alpha 2 antibody (1:1,000), Recombinant Anti-GST antibody (1:5,000) (Abcam, Cambridge, MA, USA). The specificity of the TcAK1, TcAK2, TcFOXO and TcAMPKα antibodies was verified in our previous studies [29, 58, 59] The membranes were washed and incubated with the corresponding secondary antibody such as Goat anti-mouse IgG/HRP (1:10,000) (Solarbio, Beijing, China) and Goat anti-rabbit IgG/HRP antibody (1:10,000) (Solarbio, Beijing, China). The blot signals were detected using Tanon High-sign ECL Western Blotting kit (Tanon, Shanghai, China) with a ChemiDoc Touch Imaging System (Bio-Rad, Hercules, CA, USA) as described previously [29].

### Cell culture and transfection

Sf9 cells were maintained in Sf-900 II serum-free medium (SFM) (Invitrogen, Basel, Switzerland) at 27°C. Transfection was performed with the Cellfectin II Reagent (Invitrogen, Carlsbad, USA) according to the protocol. Sf9 cells were seeded at a density of 8 × 10^5^ cells/well in 2 mL Sf-900 II SFM and cultured without antibiotics in a 6-well plate for one day. The transfected plasmids were mixed with the transfection reagents Cellfectin II at a ratio of 1:3 (mass: volume) in 100 μL Sf-900 II SFM. After incubation at room temperature for 30 min, the DNA-lipid mixture was added onto the cells dropwise. After 4–6 h, the transfection mix was replaced by a complete medium with 10% (v/v) fetal bovine serum (FBS).

HEK293 cells were cultured in high glucose Dulbecco’s modified eagle medium (DMEM) (Solarbio, Beijing, China) containing 10% (v/v) FBS (Gibco, Grand Island, NY, USA), phenol red, and 1% penicillin/streptomycin in a humidified atmosphere (5% CO_2_, 37°C). HEK293 cells were seeded (25,000 cells/cm^2^) and allowed to proliferate for 24 h during which they reached 40–60% confluence before transfection. Transfection was performed with the Lipo6000 Transfection Reagent (Beyotime, Nantong, China) according to the protocol.

### Quantitative RT-PCR (qPCR)

Total RNA was extracted from whole insects using Trizol reagent (Thermo Fisher Scientific, Waltham, MA, USA) according to the manufacturer’s protocol. cDNA was synthesized from total RNA using the PrimeScript RT reagent (Takara, Dalian, China). qPCR reactions were performed on the Bio-Rad CFX 96 Real-time PCR system using SYBR PrimeScript RT-PCR Kit II (Takara, Dalian, China) and gene-specific primers (S2 Table). The mRNA levels were normalized to the stably expressed reference gene encoding ribosomal protein S3 (rps3, GenBank: CB335975) [60] in *T*. *castaneum* and GAPDH (GenBank: NM_001256799) [61] in HEK293 cells with the 2^-ΔΔCT^ method (S2 Table) [62]. The means and standard errors for each time point were obtained from the average of at least three biologically independent sample sets. The efficiency of amplification of the primer pairs was validated before gene expression analysis (S6 Fig).

### RNA interference

The DNA templates were amplified from plasmids carrying the gene fragments with primers containing a T7 promoter listed in S2 Table. Double-stranded RNAs (dsRNAs) were synthesized using TranscriptAid T7 High Yield Transcription Kit (Thermo Fisher Scientific, Waltham, MA, USA). About 200 ng of dsRNA in 200 nL injection buffer was injected into 15-day-old larvae using a Nanoliter 2010 injector system (WPI, Sarasota, FL, USA). Target gene suppression and its downstream effector gene expressions were determined four days after larval injection. The dsEGFP-injected larvae (dsEGFP group) and the uninjected wild-type larvae (WT group) were set as controls in all injection experiments. For RNAi experiments in HEK293 cells, the synthesis of siRNA was performed using RNAi Transcription Kit (Vazyme, Nanjing, China) according to the manufacturer’s instructions with the following sequences: AMPKα1/α2, 5′-ATGATGTCAGATGGTGAATTT-3′ (targeting both AMPKα1 and α2); [63] FOXO3a, 5′-GACGATGATGCGCCTCTCT-3; CKB, 5′-GCGGTATCTGGCACAATGACAAT-3′. The irrelevant siRNA targeting EGFP (5′-GCATCAAGGTGAACTTCAA-3′) was used as control [64]. The transfections of siRNAs were performed using Lipo6000 Transfection Reagent (Beyotime, Nantong, China) according to the manufacturer’s instructions. All experiments were performed in three biological replicates.

### Construction of vectors, *in vitro* expression, and purification of recombinant proteins

For the overexpression of *TcAK1* and *TcAK2* in Sf9 cells, the ORFs of *TcAK1* and *TcAK2* were cloned into linearized pIZT/V5-His vector by *Kpn* I and *Nhe* I. Then, the Ser129 and Thr247 in TcAK1 and Ser145 in TcAK2 were replaced by aspartic acid or alanine to generate the phospho-mimetic proteins (TcAK1 S129D T247D, and TcAK2 S145D) or phospho-deficient proteins (TcAK1 S129A T247A, and TcAK2 S145A) using Mut Express MultiS Fast Mutagenesis Kit (Vazyme, Nanjing, China) and mutant primers (S2 Table), respectively. For the overexpression of CKB in HEK293 cells, the transient expression plasmids for human CKB were constructed by cloning human *CKB* ORF into the pCDNA3.1-HA vector, and the human FOXO3a overexpression vector was purchased from MiaolingBio (Wuhan, China).

For the *in vitro* phosphorylation assay, the vectors used to express the His-TcAK1 and His-TcAK2 protein in *Escherichia coli* were constructed as described previously [29]. To examine the effect of phosphorylation of TcAKs on their enzymatic activities, the site-directed mutagenesis was conducted using Mut Express MultiS Fast Mutagenesis Kit (Vazyme, Nanjing, China) and mutant primers (S2 Table) to generate TcAK1 S129D, TcAK1 T247D, or TcAK2 S145D mutant vectors that mimics phosphorylation of the TcAK1 or TcAK2. Then the correct recombinant products were transformed into *E*. *coli* M15 to express the proteins. To generate functional TcAMPKαβγ heterotrimeric complexes, we co-expressed trimeric proteins in Sf9 cells using a baculovirus expression vector [32,65]. Briefly, the ORFs of three subunits of *TcAMPK* (*TcAMPKα*, *TcAMPKβ*, and *TcAMPKγ*) were amplified separately using PCR (primers see S2 Table). The ORFs were inserted into pFastBac HTB baculovirus expression vector, respectively. The constitutively active AMPKα (T172D mutant) expression vector, according to previous reports [37], was also generated using Mut Express MultiS Fast Mutagenesis Kit (Vazyme, Nanjing, China) and mutant primers (S2 Table). The Sf9 cells were triple-infected with baculovirus and harvested by centrifugation (1000 ×g, 10 min, 4°C) at 48 h post-infection for protein purification. Purifications of the recombinant 6×His TcAMPKαβγ, 6×His TcAMPK-αT172Dβγ, 6×His TcAK1, 6×His TcAK1 S129D, 6×His TcAK1 T247D, 6×His TcAK2, and 6×His TcAK2 S145D proteins were performed using native Ni^2+^-NTA agarose chromatography (Qiagen, Valencia, CA, USA) as outlined by the manufacturer’s instructions. The enriched recombinant proteins were assayed and confirmed by SDS–PAGE and western blot. Concentrations of the purified proteins were determined using the BCA protein assay kit (Thermo Scientific, Rockford, IL, USA).

To express the GST fusion protein, the ORF of *TcAK1* or *TcAK2* was amplified from the cDNA using primers sharing 15 homologous bases at each end of vector pGEX-4T1, which was linearized with the restriction enzymes *BamH* I and *Not* I (S2 Table). The PCR products were cloned into the vector pGEX-4T1 using In-Fusion HD cloning kit (Clontech, Palo Alto, CA, USA) according to the manufacturer’s protocol. The recombinant vectors were sequenced, and the correct plasmid vectors were transformed into *E*. *coli* BL21 and induced by 1 mM isopropyl-beta-D-thiogalactoside (IPTG) at 16°C for 18 h. The induced cells were collected and treated with 0.5 mg/mL lysozyme (Sigma-Aldrich, Gallarate, Italy) on ice for 30 mins in phosphate-buffered saline with Tween 20 (PBST) buffer and then lysed by sonication using five 30 s bursts interspersed with 20 s rest periods on ice, followed by centrifugation at 12,000 rpm for 30 min at 4°C to collect the soluble and insoluble fractions.

### Paraquat, H_2_O_2_ and AICAR treatments

In the related experiments, at four days after injection of dsAMPKα, dsFOXO, dsTcAK1, or dsTcAK2 into 15-day-old larvae as described above, beetles were exposed to the filter paper surface treated with 20 mM paraquat in 5% sucrose for different time intervals, and control beetles received the same amount of 5% sucrose treatment. For the subcellular localization assay of TcFOXO, after treating wild type 7-day-old female adults with 20 mM paraquat for 12 h, beetles were collected, and midgut was dissected for immunofluorescence. To further test the role of TcAMPKα in paraquat-induced TcFOXO nuclear localization, dsTcAMPKα was injected into 2-day-old female pupae, and 7-day-old female adults were treated with 20 mM paraquat for 12 h, followed by dissection of midgut for immunofluorescence assays. In the tolerance assays, at four days after injection of dsFOXO into 15-day-old larvae, the beetles were exposed to 20 mM paraquat in 5% sucrose as described above, and the mortality rate was counted. The WT group and dsEGFP group were set as controls. Three biological replications were carried out for the experiments, and at least 50 beetles were used for each replicate.

For activation of AMPK, 20-day-old larvae were injected with 100 nL (25 ng/nL) of AICAR dissolved in 10% DMSO for 2 h, and the larvae treated with an equal amount of DMSO were used as control. For combination treatment of AICAR and paraquat, the 20-day-old larvae were treated with 100 nL (25 ng/nL) of AICAR dissolved in 10% DMSO for 24 h, followed by treatment with 20 mM paraquat in 5% sucrose for different time intervals. Each experiment was replicated three times, and 50 female adults were used for each replicate.

For *in vitro* treatment in Sf9 cells, the cells were treated with 500 μM AICAR for 24 h or treated with 10% DMSO as a control, followed by treatment with paraquat or H_2_O_2_ at a final concentration of 10 μM or 500 μM, respectively, for different time intervals, and the cells were collected for the ATP level assays. To elucidate the role of TcAK1 and TcAK2 phosphorylation in maintaining intracellular ATP content under oxidative stress, the phospho-deficient mutant vectors (TcAK1 S129A T247A, and TcAK2 S145A) were transfected into Sf9 cells and incubated for 48 h, followed by treatment with 10 μM paraquat or 500 μM H_2_O_2_ for 24 h, then collected for ATP level assays. The cells transfected with the WT TcAK1 vector (pIZT-TcAK1 WT) or WT TcAK2 vector (pIZT-TcAK2 WT) were used as the controls. For in vitro treatment of HEK293 cells, after transfection with siCKB or siAMPKα1/α2 for RNAi, or transfection with pCDNA-CKB for overexpression, cells were treated with 10 μM paraquat or 500 μM H_2_O_2_ for different time intervals. siEGFP and pCDNA3.1-control were used as a control, respectively. For AICAR treatment in HEK293 cells, after transfection with siCKB or siEGFP, respectively, the medium was treated with AICAR at a final concentration of 1 mM for 24 h as described previously, followed by treatment with 10 μM paraquat or 500 μM H_2_O_2_ for 12 h.

### Assays of ATP and ADP levels

To detect tissue ATP, 100 mg of AICAR and/or paraquat treated insects were placed in 1000 μL of lysis buffer and homogenized. The lysate was centrifuged at 12,000 ×g for 5 min at 4°C. The supernatant was transferred to a new 1.5-mL tube. According to the manufacturer’s instructions, the ATP content was determined using an ATP assay kit (Beyotime, Jiangsu, China) based on a bioluminescence technique. The protein content was determined according to the method described above at the same time. The relative ATP level was expressed as an ATP value/protein value [66]. To examine the effect of TcAK1 and TcAK2 phosphorylation on the ATP content of Sf9 cells under normal conditions, we transfected the Sf9 cells with the phospho-mimetic TcAK mutant vectors (pIZT-TcAK1 S129D T247D or pIZT-TcAK2 S145D), then the ATP and ADP contents of the cells were determined using ATP assay kit (Beyotime, Jiangsu, China) as described above and ADP Assay Kit (Colorimetric/Fluorometric) (Abcam, Cambridge, MA, USA) according to the protocol, respectively. The cells transfected with the WT TcAK1 vector (pIZT-TcAK1 WT) WT or WT TcAK2 vector (pIZT-TcAK2 WT) were used as the controls. The ATP and ADP levels were normalized for the number of cells [67], and the ratio of ATP to ADP was calculated. Each experiment was replicated three times.

### Oxidative stress tolerance assay

Insects from the dsTcFOXO-injected group were exposed to the filter paper surface treated with 20 mM paraquat in 5% sucrose for 12 h as described above, and 5% sucrose treatment was used as control. Then, the mortality rate was counted. The WT group and dsEGFP group were set as controls. Three biological replications were carried out for the experiments, and at least 50 beetles were used in each replicate treatment.

### Subcellular localization

Immunofluorescence was used to detect the subcellular localization of TcFOXO. Midguts of larvae from the corresponding treatment and control groups were dissected and washed three times with PBS, and the immunofluorescence experiments were conducted as described previously [29, 68]. Briefly, midguts of 20-day-old larvae were washed three times with PBS and then incubated in ice-cold PBS containing 4% (v/v) paraformaldehyde for two hours. After washing three times with PBS, the midguts were permeabilized by incubating for one hour with PBS containing 1% (v/v) Triton X-100 at room temperature. Nonspecific antibody binding sites were blocked by incubating with 5% bovine serum albumin (BSA) in PBS containing 0.1% (v/v) Tween-20 (PBST) for 1 h at 37°C. The dissected tissues were incubated with the antibodies raised against TcFOXO for 1 h and were washed with PBS three times. Then the tissues were incubated with Alexa Fluor 555 dye-labeled goat anti-rabbit IgG (Invitrogen, Carlsbad, CA, USA) diluted 1: 1,000 in PBS for 1 h. Then the washed tissues were mounted on a micro slide (Vector Laboratories, Burlingame, CA, USA) with the DAPI mounting medium (Solarbio, Beijing, China) to visualize the cell nucleus. Images were acquired with Leica TCS SP8 STED 3×super-resolution microscope and processed using Leica Application Suite Advanced Fluorescence software (LAS AF, Leica, Heidelberg, Germany).

To confirm the subcellular localization of the target protein that was shown in the immunofluorescence staining result, nuclear, cytoplasmic, and total proteins were extracted from corresponding control and treatment groups using the Nuclear and Cytoplasmic Protein Extraction Kit (Beyotime, Jiangsu, China) and Tissue Protein Extraction Kit (ComWin Biotech, Beijing, China) according to the manufacturer’s instructions and were quantified using a BCA Protein Assay Kit (Thermo Scientific, Rockford, IL, USA) according to the manufacturer’s instructions. Western blot experiments were conducted as described above. The anti-α-tubulin mouse monoclonal antibody (Proteintech Group, Inc., Chicago, IL, USA) was used as a loading control for the cytoplasmic and total proteins, and the anti-Histone H3 Mouse Monoclonal Antibody (Beyotime, Jiangsu, China) was used as a loading control for the nuclear protein.

### Immunoprecipitation (IP) and co-immunoprecipitation (Co-IP)

Immunoprecipitation was used to purify TcFOXO, TcAK, or TcAK2 protein from the whole-cell proteins. Proteins were extracted from the whole insects using one step animal cell active protein extraction kit (Sangon, Shanghai, China). The protein concentration was quantified using a BCA Protein Assay Kit (Thermo Scientific Pierce, IL, USA). Equal total protein amounts (100 mg) were incubated overnight at 4°C with 2 μg purified IgG against the corresponding proteins, followed by incubation with 40 μL pre-prepared solution of Protein A+G Agarose (Beyotime, Jiangsu, China) at 4°C for 3 h. The immune complex with agarose was subsequently washed five times in PBS to remove unbound protein and captured by centrifugation at 2,500 rpm for 10 min at 4°C. Finally, the immunoprecipitate was eluted with 50 μL 0.2 M glycine pH 2.6 (1:1) by incubating the sample for 10 min three times with frequent agitation before gentle centrifugation. The eluate was pooled and neutralized by adding an equal volume of Tris pH 8.0. The beads were neutralized by washing two times with 150 μL lysis buffer (without detergent) and pooled with eluate. The samples were run on SDS-PAGE, and western blot was used to check the precipitation of target proteins.

Co-immunoprecipitation was carried out to verify the interaction between TcAMPKα and TcAK1, TcAK2, or TcFOXO. The immunoprecipitation procedure was conducted with the pull-down antibody (anti-TcFOXO antibody, anti-TcAK1 antibody, or anti-TcAK2 antibody) or nonimmune rabbit IgG (control group) as described above, then the immune complex with agarose was finally resuspended in 100 μl of 1 × SDS sample buffer and denatured by boiling beads at 100°C for 5 min. Western blot was used to detect the TcAMPKα protein in the eluted samples. To compare the difference in protein content under different conditions, we added α-tubulin assay to the input group to control the consistency of the loading volume.

### Detection of phosphorylation levels

TcFOXO, TcAK1, or TcAK2 was purified by immunoprecipitation as described above for detecting phosphorylation levels after treatments with paraquat or AICAR. Concentrations of the purified proteins were determined using the BCA protein assay kit (Thermo Scientific, Rockford, IL, USA). Then, the proteins were separated on 10% SDS-PAGE and stained with Pro-Q Diamond [33,34,69] according to the manufacturer’s instructions (Invitrogen, Carlsbad, CA, USA). In brief, after electrophoresis, the gels were incubated in 100 mL of 50% methanol with 10% acetic acid overnight at room temperature and washed three times with deionized water for 10 min each time. Gels were stained with 50 mL of Pro-Q Diamond staining solution for 90 min in the dark and destained three times in 100 mL of Pro-Q Diamond destaining solution in the dark for 30 min each time and washed two times with deionized water for 5 minutes each time. The level of protein loading was determined by coomassie blue stain with Protein Stains H (Sangon, Shanghai, China) according to the manufacturer’s instructions. The gel was imaged using a ChemiDoc imaging system (Bio-Rad, Hercules, CA, USA) with default settings. The number of moles of phosphate per mole of purified protein was further determined using the Phosphoprotein Phosphate Estimation Assay Kit (Sangon, Shanghai, China), based on the alkaline hydrolysis of phosphate from seryl and threonyl residues in phosphoproteins [35, 70]. The released phosphates were quantified using malachite green and ammonium molybdate.

### Dual-luciferase reporter assay

For dual-luciferase reporter assays, the upstream regulatory regions of TcAK1 (from -2170 to +130 bp) and TcAK2 (from -2079 to +88 bp) were amplified by PCR from genomic DNA, then subcloned into the linearized pGL-Basic3.1 reporter vector by restriction enzymes *Kpn* I and *Nhe* I. Site-directed mutagenesis of TcFOXO binding site with the highest score predicted by JASPAR database (http://jaspar.genereg.net/) in the promoter regions of TcAK1 (TTGTAAACACGC to ACCAGGGATGTT, position -1307–-1296 bp) and TcAK2 (TTGTAAATATTT to TCTGCTGTTTAG, position -636–-625 bp) was performed as described above (S2 Table). For overexpression of TcFOXO in the Sf9 cells, the ORF of *TcFOXO* was amplified from cDNA using primers sharing 15 homologous bases at each end of the vector pIZT/V5-His linearized with the restriction enzymes *Kpn* I and *Nhe* I (S2 Table) and then cloned into the linearized vector pIZT/V5-His using the In-Fusion HD cloning kit (Clontech, Palo Alto, CA, USA) as described above. The transfection of Sf9 cells was performed as described above. Briefly, 1 μg pGL-TcAKs or pGL-Control and 0.5 μg pIZT-TcFOXO were co-transfected into the cells and 0.1 μg pIZT-RLuc vector containing the *Renilla* luciferase gene constructed as previously described was used as the reference for insect cells [71]. The relative luciferase activity in each sample was determined at 48 h post-transfection using the dual-Luciferase reporter assay system (Promega, Madison, WI, USA) according to protocol and analyzed with GloMax 20/20 luminometer (Promega, Madison, WI, USA). All reporter assays were repeated three times (n = 3) independently, and the average expression levels of the report genes were represented as mean ± SEM.

### Chromatin immunoprecipitation assay (ChIP)

ChIP was performed as described previously with slight modifications [72,73]. Briefly, about 100 mg larvae treated with paraquat for 8 h or AICAR for 2 hours were frozen and homogenized in Nuclear Isolation buffer (10 mM MOPS; 5 mM KCl; 10 mM EDTA; 0.6% Triton X-100) containing protease and phosphatase inhibitor cocktail (Beyotime, Shanghai, China). The homogenized tissue was centrifuged at 800 rpm for 1 min at 4°C and the pallet was discarded. Formaldehyde in a final concentration of 1% was added to the supernatant, followed by incubation at room temperature for 10 min and the addition of 0.136 M glycine. Nuclei were pelleted by centrifugation and resuspended in Nuclear Lysis buffer (50 mM Tris-HCl pH 7.7; 1% SDS; 20 mM EDTA) and sonicated eight times for each 20 s pulses on ice, followed by centrifugation at 15,000 rpm for 3 min at 4°C. Then, 10% of the sample was removed for an input control chromatin, and 100 μL for quantification. The reversal of cross-link was performed by the addition of 20 mM EDTA and 0.2 M NaCl and incubation overnight at 65°C. The material was digested with proteinase K (200 μg/ml) and RNase A (50 μg/ml) overnight at 37°C, and the DNA was extracted by phenol: chloroform: isoamyl alcohol (25:24:1). The DNA concentration was measured by NanoPhotometer (Implen, Munich, Germany). The sonicated supernatant containing 25 μg of chromatin was subsequently diluted 10-fold with ChIP dilution buffer (1% Triton X-100; 10 mM EDTA; 15 mM Tris-HCl pH 7.5; 300 mM NaCl) for subsequent immunoprecipitation. The supernatant was incubated overnight at 4°C with 2 μg anti-TcFOXO antibody or 2 μg nonimmune rabbit IgG control and followed by incubation with 40 μL pre-prepared solution of Protein A+G Agarose (Beyotime, Shanghai, China) at 4°C for 3 h. The immune complex with agarose was subsequently washed five times in ChIP dilution buffer, and captured by centrifugation for 10 min at 15,000 rpm and 4°C. After reverse cross-link and proteinase K (200 μg/ml) and RNase A (50 μg/ml) digestion, DNA was extracted as described above. qPCR was used to quantify the amount of *TcAK1* and *TcAK2* promoter fragments precipitated with antibody against FOXO. The enrichment relative to input DNA was calculated. The fold difference between the experimental sample and the negative control (IgG) was determined by 2^-ΔΔCt^ method. Primers were designed based on the predicted *TcFOXO* binding sites of *TcAK1* promoter region (position -1307–-1296 bp) or *TcAK2* promoter region (position -636–-625 bp) (S2 Table).

### Yeast two-hybrid assay

Interactions of TcAK1 or TcAK2 with TcAMPKα WT or constitutively active TcAMPKα T172D mutant were analyzed using the Matchmaker Gold Yeast Two-Hybrid System (Takara, Dalian, China). Briefly, the ORFs of *TcAK*s were cloned into the pGADT7 vector (prey), while *TcAMPKα* WT subunit was cloned into the pGBKT7 vector (bait). The constitutively active TcAMPKα T172D mutant vector was constructed as described above. The *Saccharomyces cerevisiae* strain Y2HGold was co-transformed with bait and prey plasmids using the lithium acetate method. Interaction of proteins activates reporter gene transcription, which is indicated by the growth of yeast cells on nutrient-deficient medium. Yeast cells were spotted on selective medium lacking tryptophan and leucine (SD-WL) to verify the presence of bait and prey plasmid, and on medium lacking adenine, histidine, tryptophan, and leucine (SD-AHWL) for protein interaction analysis. Spotted plates were incubated for 72 h at 30°C. The activation of reporter genes due to protein interactions was detected by adding x-α-gal to yeast strains through the *MEL1* reporter gene.

### GST pull-down assay

GST pull-down was conducted using the Mag-Beads GST Fusion Protein Purification (Sangon, Shanghai, China) according to the manufacturer’s instructions. Briefly, the GST-protein lysate was added to the glutathione agarose beads and mixed by inversion and incubated at 4°C for 1 h. The target protein magnetic bead complex was washed three times with binding buffer/washing buffer (140 mM NaCl, 2.7 mM KCl, 10 mM Na_2_HPO_4_, 1.8 mM KH_2_PO_4_, pH 7.4). The purified GST-TcAK1, GST-TcAK2 fusion, or GST protein magnetic bead complex was incubated with 1μg of protein extracted from larvae in the pull-down buffer (140 mM NaCl, 2.7 mM KCl, 10 mM Na_2_HPO_4_, 1.8 mM KH_2_PO_4_, pH 7.4) at 4°C for 1 h, respectively. The target protein magnetic bead complexes were washed using the pull-down buffer for three times. SDS-PAGE buffer was added to the protein complexes and boiled. Then, the proteins were separated on 10% SDS-PAGE, transferred on a PVDF membrane, and detected by western blot using anti-GST antibody or Mouse Anti-AMPK alpha 1 + AMPK alpha 2 antibody (Abcam, Cambridge, MA, USA).

### *In vitro* phosphorylation assay, MALDI TOF/TOF–MS/MS and LC-MS/MS

*In vitro* phosphorylation of TcAK1 and TcAK2 was carried out as described previously [37]. Briefly, 240 pmol 6×His TcAK1 or 6×His TcAK2 recombinant protein was incubated for 60 min at 37°C in the presence of 10 pmol TcAMPKαT172Dβγ protein in kinase buffer (pH 7.4) containing 200 μM ATP, 50 μM AMP, 5 mM MgCl_2_, 1 mM DTT, 10 mM HEPES and protease and phosphatase inhibitor cocktail (Beyotime, Shanghai, China). Kinase reactions were stopped by the addition of SDS buffer. TcAKs proteins in the reaction system were then purified using Protein A+G Agarose (Beyotime, Shanghai, China) and anti-TcAK1 antibody or anti-TcAK2 antibody according to the manufacturer’s instructions. Phosphopeptide analysis was performed by Matrix-assisted laser desorption/ionization time-of-flight-time-of-flight (MALDI TOF/TOF) tandem mass spectrometry (MS/MS) as described previously [74]. Briefly, the purified proteins were enzymatically digested by trypsin (20 h, 37°C) and selectively enriched phosphopeptides were enriched using titanium dioxide chromatography (TiO_2_ Mag-Sepharose, GE Healthcare, Madison, WI, USA) according to the manufacturer’s instructions. The phosphopeptide eluents were vacuum-dried and dissolved in the matrix solution consisted of 9 mg/ml 2′, 4′, 6′-trihydroxyacetophenone monohydrate (THAP) and 5 mg/ml diammonium citrate (DAC) dissolved in 50:50 water/acetonitrile (v/v). Mass spectra were acquired in a 5800 Plus MALDI TOF/TOF analyzer mass spectrometer (Applied Biosystems, Framingham, MA, USA) in positive ion reflector and MS/MS modes. For LC-MS/MS analysis, the enriched peptides were fractionated using the Thermo Scientific Pierce high pH Reversed-Phase Peptide Fractionation Kit (Thermo Scientific, Rockford, IL, USA) according to the manufacturer’s instructions. Each fraction was resuspended in 20 μL buffer A (2% acetonitrile, 0.1% formic acid) and centrifuged at 20,000g for 10 min. The supernatant was loaded on an UltiMate 3000 UHPLC (Thermo Scientific, Waltham, MA, USA) by the autosampler onto a trap column and subjected to desalting. Then, the peptides were eluted onto a C18 analytical column (15 cm × 75 μm) with a linear gradient 5–80% buffer B (98% acetonitrile, 0.1% formic acid) over 95 min at a flow rate of 300 nL /min. The separated peptides were ionized by the nanoESI source and injected into a Q-Exactive PLUS tandem mass spectrometer (Thermo Fisher Scientific, San Jose, CA, USA) under data-dependent acquisition (DDA) mode. Detection of intact peptides and ion fragments was performed in the Orbitrap at a resolution of 70,000 and 17,500, respectively. Peptides were selected for MS/MS using high-energy collision dissociation (HCD) operating mode. The electrospray voltage was set to 2.0 kV. Automatic gain control (AGC) target was set to 3e6 for full MS and 1e5 for MS2. MS/MS spectra were analyzed with the help of Proteome Discoverer 2.5 integrated with Mascot 2.3 (Thermo Fisher Scientific, San Jose, CA) and searched against *T*. *castaneum* protein sequences from the UniProt database (18675 entries, downloaded in June 2020). A strict 1% false discovery rate (FDR) threshold was set to filter the peptides using the target-decoy strategy [75]. Only phosphosites with more than 0.75 phsophoRS scores were considered as highly reliable phosphosites [76].

### Bimolecular fluorescence complementation (BiFC) assay

BiFC assay was conducted to determine the potential interactions between TcFOXO and TcAMPKα according to the previous study [77, 78]. To construct the vectors used for the BiFC assays, the GFP of pIZT/V5-His was split between residues 157 and 158 using reverse PCR. The N- and C-terminal fragments were fused with the full-length coding sequences of *TcFOXO* and *TcAMPKα*, respectively. Sf9 cells were cultured on coverslips in 24-well plates and cotransfected with plasmid pairs expressing Nt-GFP-TcFOXO and Ct-GFP-TcAMPKα fusion proteins. At 48 h post-transfection, the protein expression of TcFOXO and TcAMPKα was verified using western blot. The cells were then visualized using a fluorescence microscope (Nikon Eclipse Ts2R, Nikon, Tokyo, Japan) to observe the BiFC signal. All the assays were repeated independently three times with comparable results.

### *In vivo* assay of AK activities

AK activity assays were performed as described previously [79]. The concentrations of purified 6 × His TcAK1 WT, 6 × His TcAK1 T247D, 6 × His TcAK1 S129D, 6 × His TcAK2 S145D or immunoprecipitated TcAK1 and TcAK2 proteins were determined using a BCA protein assay kit (Thermo Scientific Pierce, IL, USA) according to the manufacturer’s protocol. To determine the K_m_ value toward ATP, a 0.2 mL reaction mixture was prepared by combining 50 mM Tris–HCl, pH 7.5, 4 mM DTT, 2 units/mL pyruvate kinase, 20 mM MgCl_2_, 0.9 mM ATP, 2.5 mM phosphoenolpyruvate, 0.2 mM NADH, and 1 unit/mL lactate dehydrogenase. After the addition of TcAK1 or TcAK2, the reaction was started with an addition of 5 mM L-arginine (final concentration). The Michaelis–Menten constant (K_m_) and catalytic rate constant (k_cat_) for ATP were determined based on different concentrations of ATP. To determine the K_m_ value for ADP, the reaction mixture contained 50 mM Tris–HCl pH 7.5, 20 mM MgCl_2_, 10 mM glucose, 2 mM nicotinamide adenine dinucleotide phosphate (NADP^+^), 4 mM DTT, 2 units/mL hexokinase, 1 unit/mL glucose-6-phosphate dehydrogenase, and 5 mM phospho-L-arginine, followed by adding TcAK1 or TcAK2 and different concentrations of ADP. All reactions were carried out at 30°C in four replications. Absorbance was measured at 340 nm to obtain the initial velocity values, and a Lineweaver–Burk plot was made and fitted by the least-square method to calculate kinetic constants (K_m_ and k_cat_).

### Cell counting Kit-8 cell viability assay

The phospho-deficient vectors (TcAK1 S129A T247A, and TcAK2 S145A) were transfected into Sf9 cells separately as described above, followed by treatment with paraquat (10 μM) for 12 h, 24 h, 36 h, and 48 h. The cells transfected with the WT TcAK1 vector (pIZT-TcAK1 WT) WT or WT TcAK2 vector (pIZT-TcAK2 WT) were used as the controls. The cells were resuspended, and 200 μL of medium was taken for the assay. Then, the cells were mixed with 10 μL of Cell Counting Kit-8 (CCK-8) solutions (Beyotime, Jiangsu, China) per well and incubated for 2 h at 37°C. The amount of formazan dye generated by cellular dehydrogenase activity was measured as absorbance at 450 nm. The optical density values of each well represented the survival/proliferation of Sf9 cells.

### Statistical analysis and reproducibility

All experiments were performed in at least three independent biological replicates and at least 30 insects were used per replicate. Data are presented as mean values ± standard error of the mean (SEM). Student’s t-test was used for comparing two means and ANOVA with post hoc Tukey’s HSD test was used for multiple comparisons of parametric data. All statistical analyses were performed using SPSS software (SPSS 13.0 for windows; SPSS Inc., Chicago, IL, USA). For the t-test: *, p<0.05; **, p<0.01. ANOVA: bars labeled with different lowercase letters are significantly different (p<0.05).

## Supporting information

S1 Fig Comparison of residues in PKs of diverse organisms corresponding to phosphorylation sites in TcAK1 and TcAK2. Amino acids sequences are obtained from the following GenBank entries: XP_021697332 for *Aedes aegypti* AK2; XP_001657389 for *A*. *aegypti* AK2; NP_729446 for *Drosophila melanogaster* AK1; NP_001011603 for *Apis mellifera* AK1; XP_026301800 for *A*. *mellifera* AK2; AEV23883 for *Blattella germanica* AK; XP_021202112 for *Bombyx mori* AK; XP_030031664 for *Manduca sexta* AK; AGH14262 for *Spodoptera frugiperda* AK; AAC82390 for *Trypanosoma cruzi* AK; NP_507054 for *Caenorhabditis elegans* AK; Q9U9J4 for *Carcinus maenas* AK; AKG50107 for *Cherax quadricarinatus* AK; AID47194 for *Marsupenaeus japonicus* AK; BAD11950 for *Crassostrea gigas* AK; NP_001301013 for *Limulus polyphemus* AK; NP_001814.2 for *Homo sapiens* CKB; NP_001815.2 for *H*. *sapiens* CKM.
(TIFF)

S2 Fig Co-IP assay for detection of TcAMPK–TcFOXO interaction under paraquat treatment. Co-IP assays were performed using the anti-TcFOXO antibody, then the immune complex with agarose was subjected to western blot analysis to detect the presence of TcAMPKα protein with the anti-TcAMPKα antibody. The whole-cell lysates (input) were also tested with either anti-TcFOXO (WB: TcFOXO) or anti-TcAMPKα (WB: TcAMPKα) antibodies to verify the protein expression, and α-tubulin was used as a loading control.
(PDF)

S3 Fig Alignment of the AMPK consensus phosphorylation motif and phosphorylation sites in *C*. *elegans* DAF-16, *H*. *sapiens* FOXO3, and TcFOXO. Amino acids sequences are obtained from the following GenBank entries: NP_001367893 for *C*. *elegans* DAF-16; AAH21224 for *H*. *sapiens* FOXO3. Phosphorylation sites are marked with red arrows; ϕ: hydrophobic residues (Blue box); β: basic residues (Black box).
(PDF)

S4 Fig Co-IP assay for detection of TcAMPK–TcAKs interaction under paraquat treatment. Co-IP assays were performed using an anti-TcAK1 or TcAK2 antibody, then the immune complex with agarose was subjected to western blot analysis to detect the presence of TcAMPKα protein with the anti-TcAMPKα antibody. The whole-cell lysates (input) were also tested with anti-TcAK1 (WB: TcAK1), anti-TcAK2 (WB: TcAK2), or anti-TcAMPKα (WB: TcAMPKα) antibody to verify the protein expression, and α-tubulin was used as a loading control.
(PDF)

S5 Fig AMPK-FOXO-CKB pathway is involved in the maintenance of ATP homeostasis in H_2_O_2_-treated HEK293 cells. **(a)** Knockdown of *CKB* or *AMPKα1*/*α2* mRNA expression increased the fold of ATP decrease caused by H_2_O_2_ treatment for 24 h in HEK293 cells. **(b)** Knockdown of *CKB* mRNA expression attenuated the AICAR-induced increase in ATP content under H_2_O_2_ treatment in HEK293 cells. **(c)** Overexpression of CKB significantly rescued the reduction of ATP content caused by H_2_O_2_ treatment in HEK293 cells. **(d)** Knockdown of *AMPKα1*/*α2* mRNA expression attenuated the H_2_O_2_-induced mRNA expression of *CKB* in HEK293 cells. **(e)** Knockdown of *FOXO3a* attenuated the H_2_O_2_-induced mRNA expression of *CKB* in HEK293 cells. mRNA expression was determined by qPCR using GAPDH as an internal reference. Data were expressed as mean ± SEM (n = 3 biologically independent replicates). Asterisks indicate differences statistically significant at * *P* < 0.05 (student’s t-test).
(TIFF)

S6 Fig The amplification efficiencies of primers for qPCR. The standard curve analysis showed that the amplification efficiency of primers was 104.8% for TcAK1 **(a)**, 105.0% for TcAK2 **(b)**, 99.9% for TcFOXO **(c)**, and 109.1% for TcAMPKα**(d)**.
(PDF)

S1 Table The predicted TcFOXO binding sites in the promoter regions of *TcAK1* and *TcAK2*. (DOCX)

S2 Table Oligonucleotide primers. (DOCX)

S1 File Numerical data underlying graphs. (XLSX)
