# Supplementary figures and images for: Restoration of energy homeostasis under oxidative stress: Duo synergistic AMPK pathways regulating arginine kinases
Source: PLoS Genet. 2023 Aug 3;19(8):e1010843. doi: 10.1371/journal.pgen.1010843 (PMC10427004; doi:10.1371/journal.pgen.1010843)

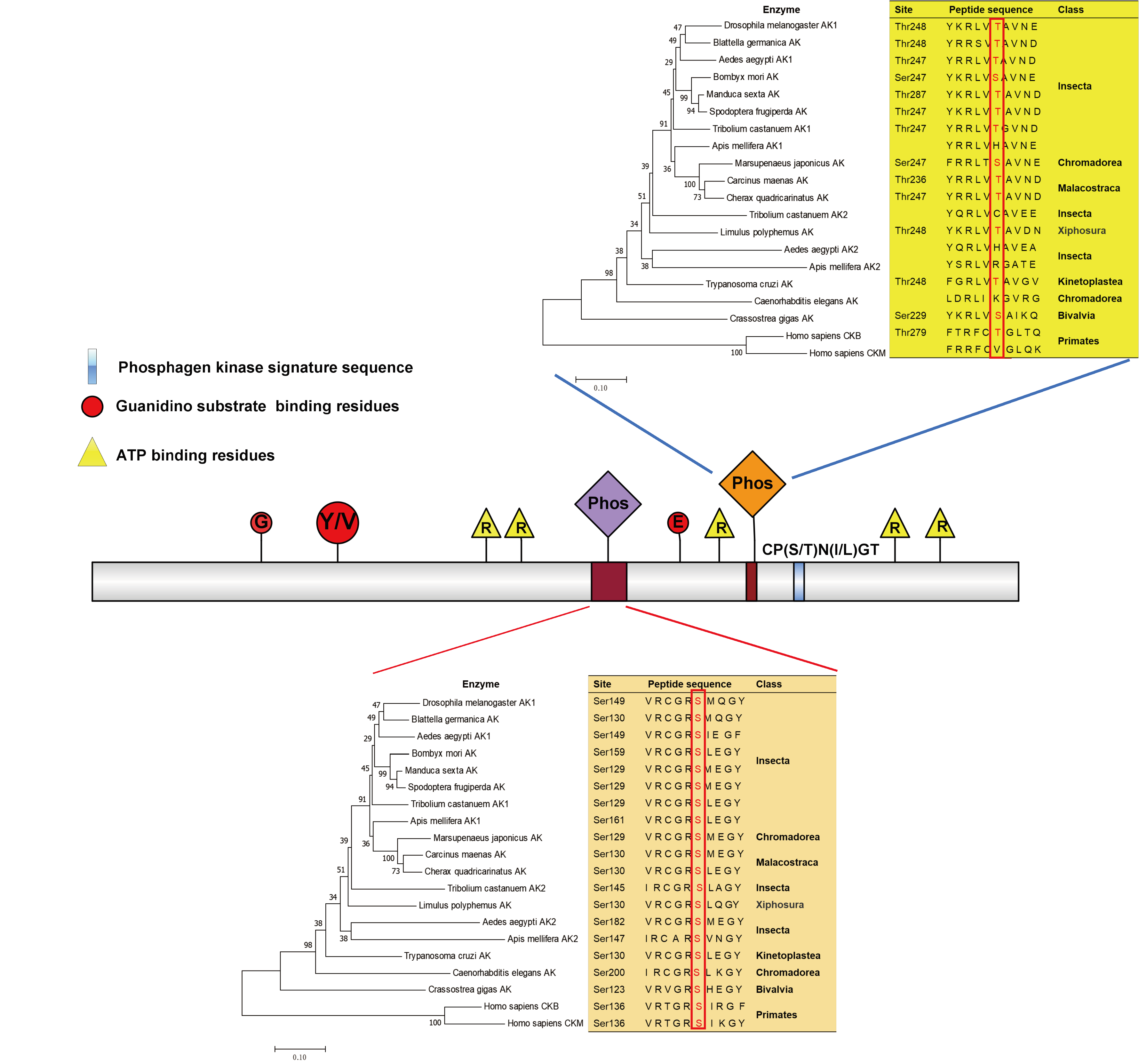

Supplement: S1 Fig — Amino acids sequences are obtained from the following GenBank entries: XP_021697332 for Aedes aegypti AK2; XP_001657389 for A. aegypti AK2; NP_729446 for Drosophila melanogaster AK1; NP_001011603 for Apis mellifera AK1; XP_026301800 for A. mellifera AK2; AEV23883 for Blattella germanica AK; XP_021202112 for Bombyx mori AK; XP_030031664 for Manduca sexta AK; AGH14262 for Spodoptera frugiperda AK; AAC82390 for Trypanosoma cruzi AK; NP_507054 for Caenorhabditis elegans AK; Q9U9J4 for Carcinus maenas AK; AKG50107 for Cherax quadricarinatus AK; AID47194 for Marsupenaeus japonicus AK; BAD11950 for Crassostrea gigas AK; NP_001301013 for Limulus polyphemus AK; NP_001814.2 for Homo sapiens CKB; NP_001815.2 for H. sapiens CKM. (TIFF) [file pgen.1010843.s001.tiff]

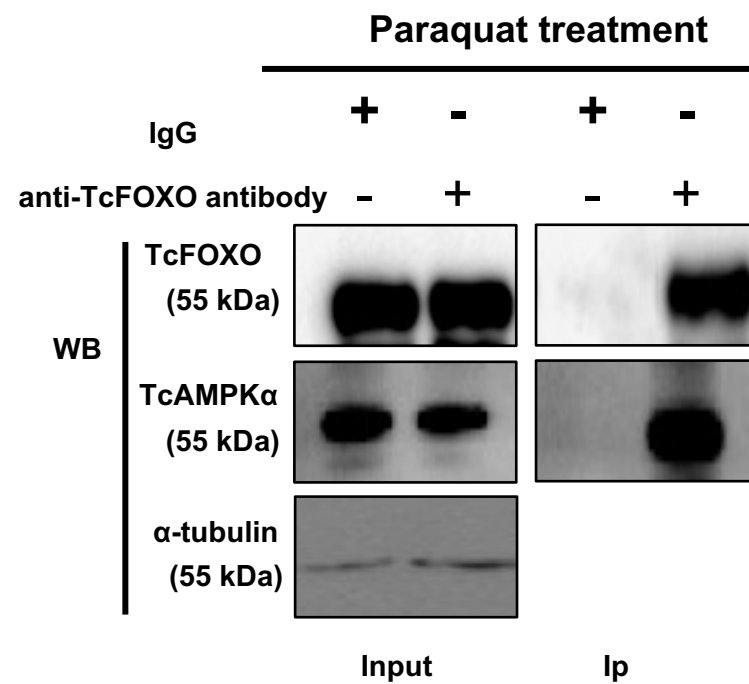

Supplement: S2 Fig — Co-IP assays were performed using the anti-TcFOXO antibody, then the immune complex with agarose was subjected to western blot analysis to detect the presence of TcAMPKα protein with the anti-TcAMPKα antibody. The whole-cell lysates (input) were also tested with either anti-TcFOXO (WB: TcFOXO) or anti-TcAMPKα (WB: TcAMPKα) antibodies to verify the protein expression, and α-tubulin was used as a loading control. (PDF) [file pgen.1010843.s002.pdf]

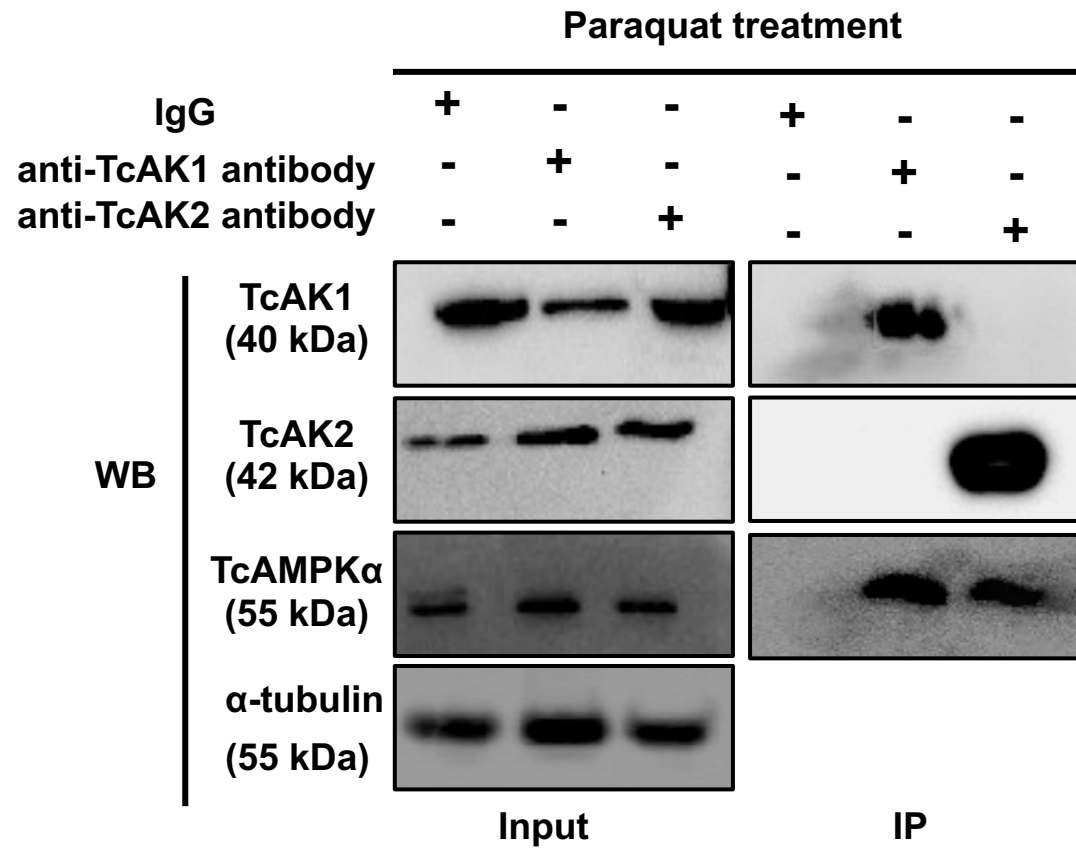

Supplement: S4 Fig — Co-IP assays were performed using an anti-TcAK1 or TcAK2 antibody, then the immune complex with agarose was subjected to western blot analysis to detect the presence of TcAMPKα protein with the anti-TcAMPKα antibody. The whole-cell lysates (input) were also tested with anti-TcAK1 (WB: TcAK1), anti-TcAK2 (WB: TcAK2), or anti-TcAMPKα (WB: TcAMPKα) antibody to verify the protein expression, and α-tubulin was used as a loading control. (PDF) [file pgen.1010843.s004.pdf]

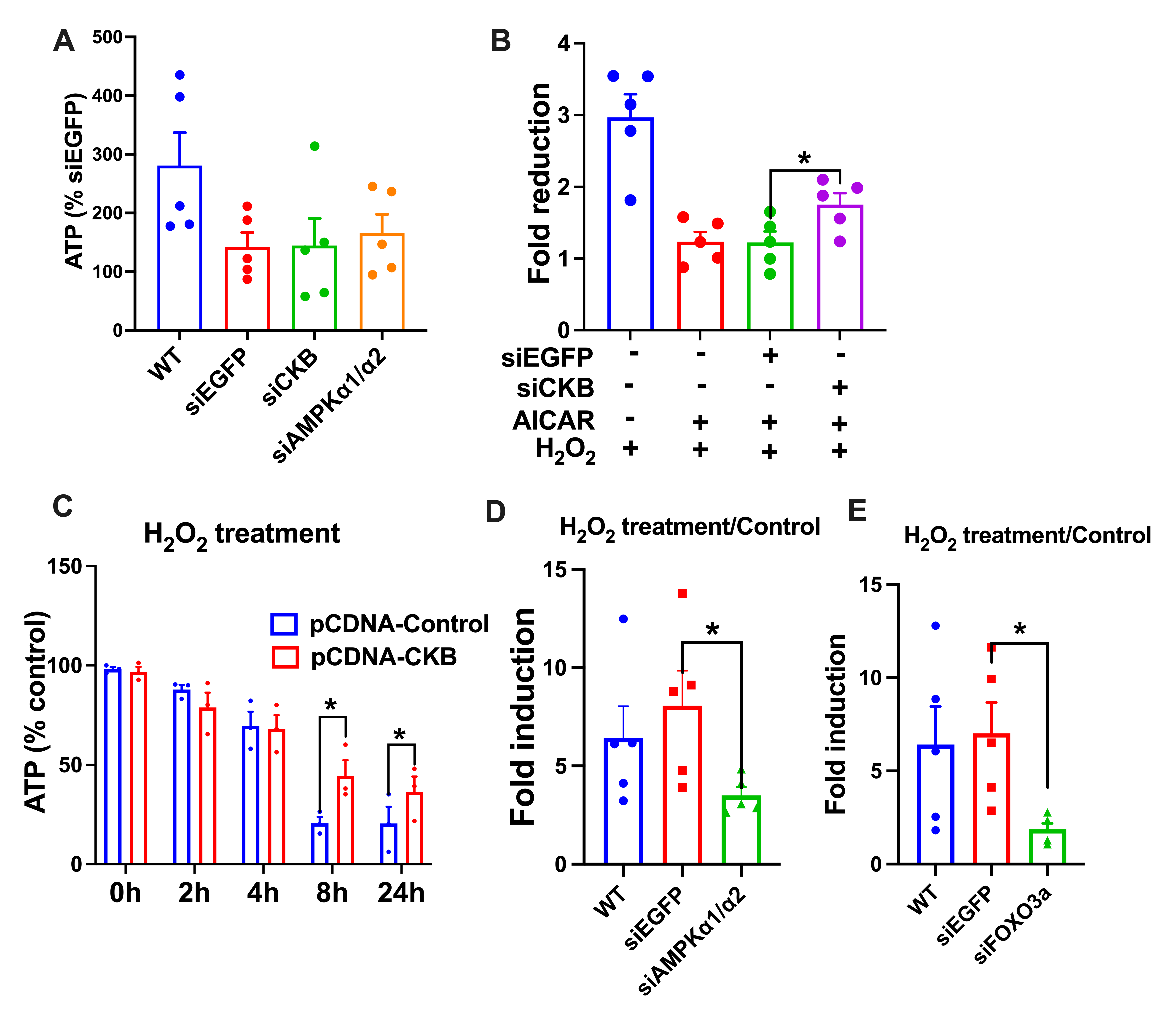

Supplement: S5 Fig — (a) Knockdown of CKB or AMPKα1/α2 mRNA expression increased the fold of ATP decrease caused by H2O2 treatment for 24 h in HEK293 cells. (b) Knockdown of CKB mRNA expression attenuated the AICAR-induced increase in ATP content under H2O2 treatment in HEK293 cells. (c) Overexpression of CKB significantly rescued the reduction of ATP content caused by H2O2 treatment in HEK293 cells. (d) Knockdown of AMPKα1/α2 mRNA expression attenuated the H2O2-induced mRNA expression of CKB in HEK293 cells. (e) Knockdown of FOXO3a attenuated the H2O2-induced mRNA expression of CKB in HEK293 cells. mRNA expression was determined by qPCR using GAPDH as an internal reference. Data were expressed as mean ± SEM (n = 3 biologically independent replicates). Asterisks indicate differences statistically significant at * P < 0.05 (student’s t-test). (TIFF) [file pgen.1010843.s005.tiff]

A

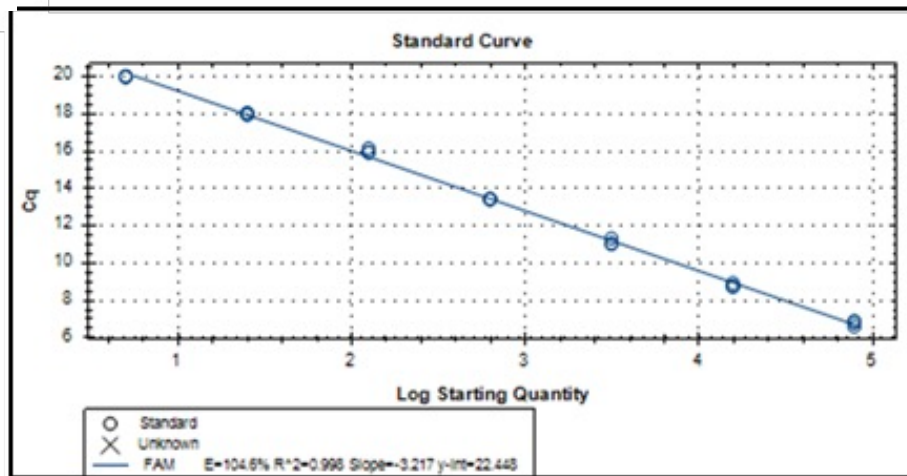

TcAK1

B

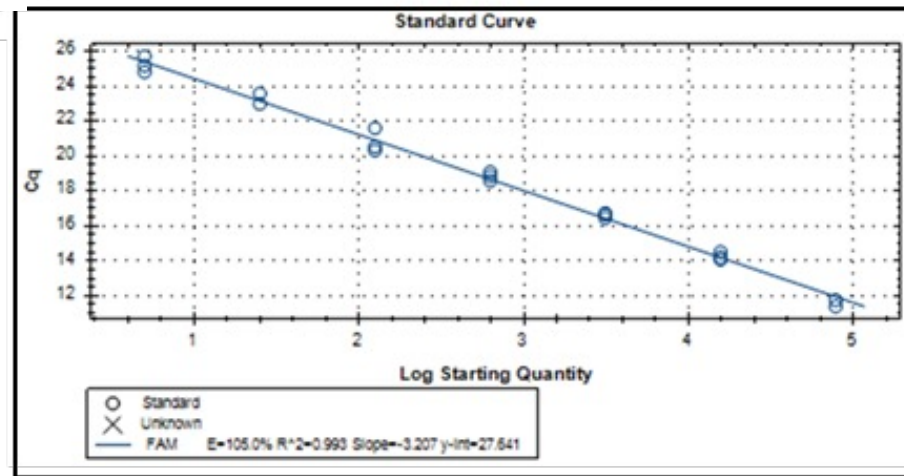

TcAK2

C

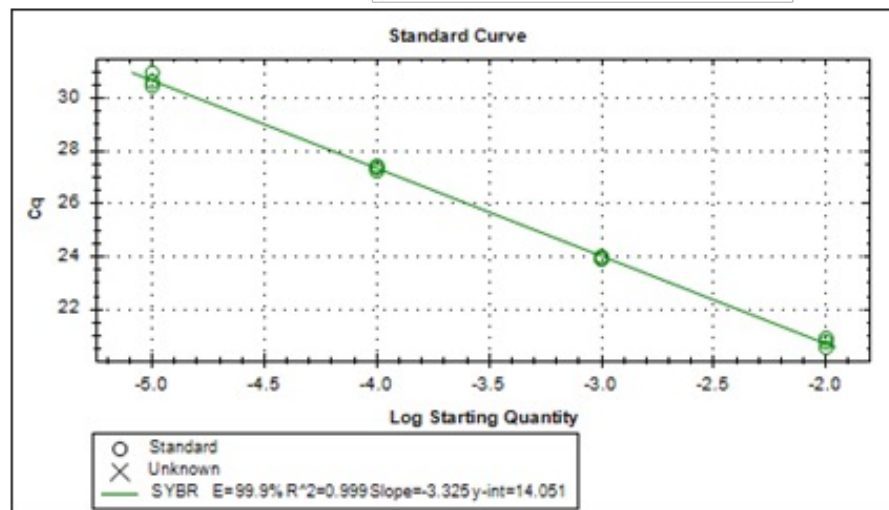

TcFOXO

D

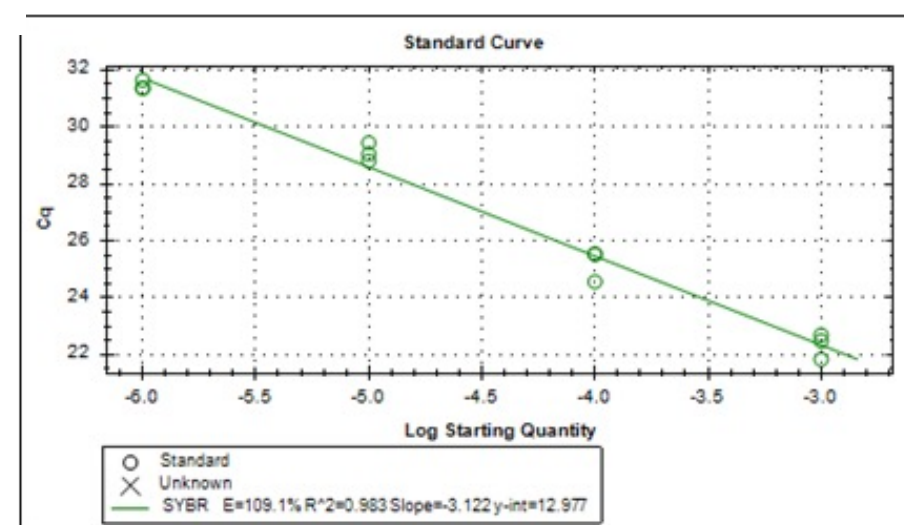TcAMPK $\alpha$

Supplement: S6 Fig — The standard curve analysis showed that the amplification efficiency of primers was 104.8% for TcAK1 (a), 105.0% for TcAK2 (b), 99.9% for TcFOXO (c), and 109.1% for TcAMPKα(d). (PDF) [file pgen.1010843.s006.pdf]
