# Supplementary material for: Restoration of energy homeostasis under oxidative stress: Duo synergistic AMPK pathways regulating arginine kinases
Source: PLoS Genet. 2023 Aug 3;19(8):e1010843. doi: 10.1371/journal.pgen.1010843 (PMC10427004; doi:10.1371/journal.pgen.1010843)
Supplement: S3 Fig — Amino acids sequences are obtained from the following GenBank entries: NP_001367893 for C. elegans DAF-16; AAH21224 for H. sapiens FOXO3. Phosphorylation sites are marked with red arrows; ϕ: hydrophobic residues (Blue box); β: basic residues (Black box). (PDF) [file pgen.1010843.s003.pdf]

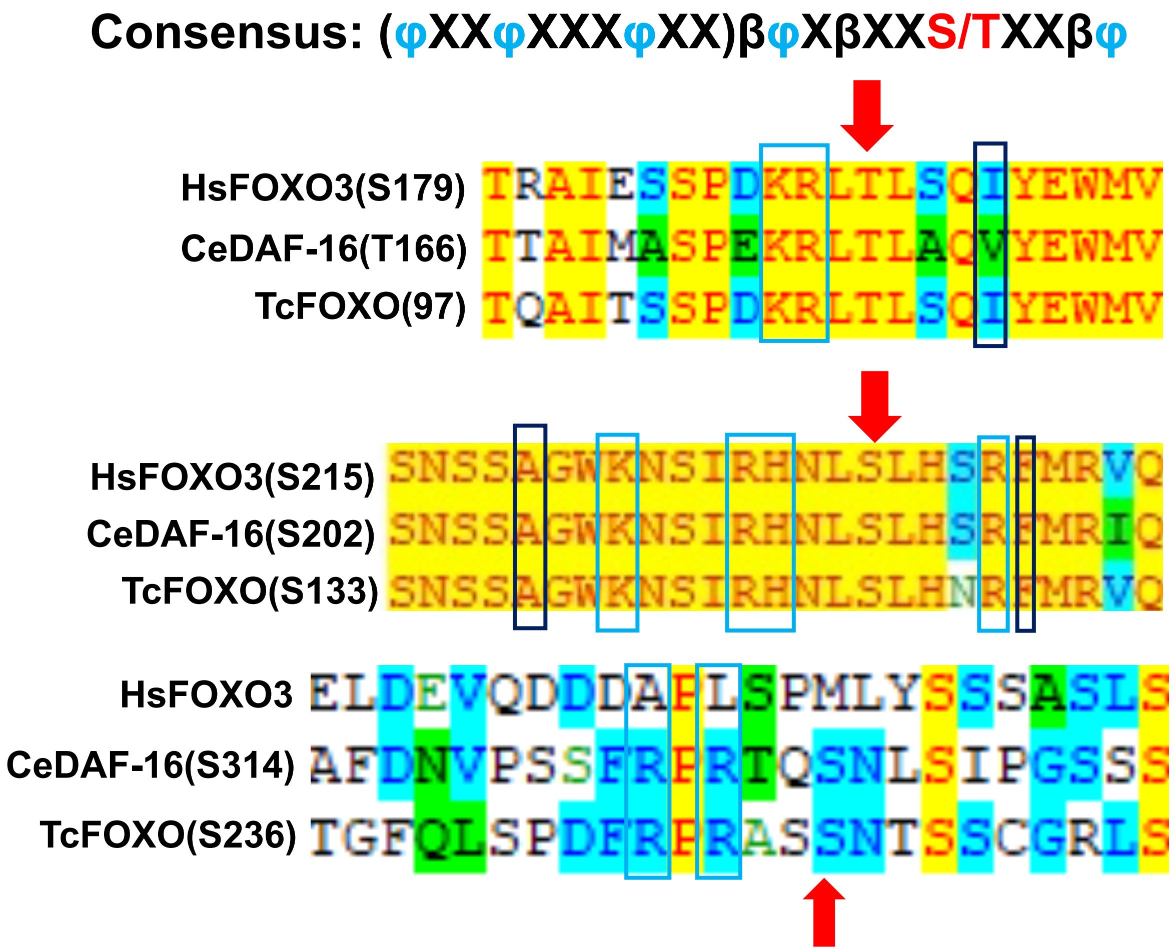
Consensus: (φXXφXXXφXX)βφXβXXS/TXXβφ
HsFOXO3(S179) TRAIESSPDKRLTLSQIYEWMV
CeDAF-16(T166) TTAIMASPEKRLTLAQVYEWMV
TcFOXO(97) TQAITSSPDKRLTLSQIYEWMV
HsFOXO3(S215) SNSSAGWKNSIRHNLSLHSRFMRVQ
CeDAF-16(S202) SNSSAGWKNSIRHNLSLHSRFMRIQ
TcFOXO(S133) SNSSAGWKNSIRHNLSLHNRFMRVQ
HsFOXO3 ELDEVQDDDAPLSPMLYSSSASLS
CeDAF-16(S314) AFDNVPSSFRPRTQSNLSIPGSSS
TcFOXO(S236) TGFQLSPDFRPRASSNTSSCGRLS
