# Supplementary material for: Restoration of energy homeostasis under oxidative stress: Duo synergistic AMPK pathways regulating arginine kinases
Source: PLoS Genet. 2023 Aug 3;19(8):e1010843. doi: 10.1371/journal.pgen.1010843 (PMC10427004; doi:10.1371/journal.pgen.1010843)
Supplement: S1 Table — (DOCX) [file pgen.1010843.s007.docx]

# S1 Table The predicted TcFOXO binding sites in the promoter regions of *TcAK1* and *TcAK2*

| **Gene** | **Position** | | | | **Strand** | | **Sequence** | |
| --- | --- | --- | --- | --- | --- | --- | --- | --- |
|  | **From** | | **To** | |  |  |  |  |
| *TcAK1* | -1349 | -1338 | | + | | TTGTAAACACGC | |  |
|  | -1002 | -991 | | + | | ACATAAACACGG | |  |
|  | -682 | -671 | | + | | TTGTAAAGACAG | |  |
|  | -1813 | -1801 | | + | | CGGTAAATATAA | |  |
| *TcAK2* | -783 | -772 | | + | | TTGTAAATATTT | |  |
|  | -114 | -103 | | + | | CAGCAAATAAAT | |  |
