# Supplementary material for: Restoration of energy homeostasis under oxidative stress: Duo synergistic AMPK pathways regulating arginine kinases
Source: PLoS Genet. 2023 Aug 3;19(8):e1010843. doi: 10.1371/journal.pgen.1010843 (PMC10427004; doi:10.1371/journal.pgen.1010843)
Supplement: S2 Table — (DOCX) [file pgen.1010843.s008.docx]

# S2 Table Oligonucleotide primers

| **Primer name** | **Sequence (5' to 3')** | **Description** |
| --- | --- | --- |
| TcAK1q-F | actaccatggcggtttcaag | TcAK1 qRT-PCR |
| TcAK1q-R | ctccttgtactgctcctcgg |  |
| TcAK2q-F | aaaacagaccaacaccctgc | TcAK2 qRT-PCR |
| TcAK2q-R | aaagtccccgacaccttctt |  |
| TcFOXOq-F | TCAGACCTCGAGCTTCCTCA | TcFOXO qRT-PCR |
| TcFOXOq-R | TGCAAGCTGATCGGGTGAAT |  |
| TcAMPKαq-F | ACATCCTTGGCCAGACTTTG | TcAMPKα qRT-PCR |
| TcAMPKαq-R | GCCGAAACAGCTTCAAGTTC |  |
| Tcrps3q-F | ACCGTCGTATTCGTGAATTGAC | rps3 qRT-PCR |
| Tcrps3q-R | ACCTCGATACACCATAGCAAGC |  |
| TcAK1Ri-F | ACAGCGACAGGTTCGGGTTC | TcAK1 dsRNA |
| TcAK1Ri-R | TTTCGGAAATGCCATCGTAC |  |
| TcAK2Ri-F | ATCGGTTTGGTTACTTGACT | TcAK2 dsRNA |
| TcAK2Ri-R | CTTTGACGGCATCATACTCC |  |
| TcFOXORi-F | TCGCCGATACCAGCAGTTGT | TcFOXO dsRNA |
| TcFOXORi-R | TGCCCTGTTCGAGGTTTCCT |  |
| NtGFP-TcFOXO-F | **tcacacaatgtatac**ATGGACTGCTACAAGATGAACCTCA | Overexpression of TcFOXO for BiFC in Sf9 |
| NtGFP-TcFOXO-R | **caacttggcatccat**ATGCACCCACGACGGC |  |
| TcAMPKαRi-F | GTTCGGGGGTTGATTACTGCC | TcAMPKα dsRNA |
| TcAMPKαRi-R | GGACTTCCTTCTCCTGCACCC |  |
| TcAK1-promoter-F-WT**^a^** | **ctatcgataggtaccgagctc**GTACAACGAACTAAAAAAGTAAAATAACTAACA | Promoter of TcAK1 |
| TcAK1-promoter-R-WT**^a^** | **gctagcttgatgccattcatg**TGCTAGAGATTAAGGGCTCAC |  |
| TcAK1-promoter-F-Mt**^b^** | TAaccagggatgttAAACATCTCCGAGAATCGGGA | Mutant of promoter of TcAK1 |
| TcAK1-promoter-R-Mt**^b^** | GTTTaacatccctggtATATTTGGGTAATAACTGGAGTCATACAA |  |
| TcAK2-promoter-F-WT**^a^** | **ctatcgataggtaccgagctc**ACAGAGACAATTAAATAAATAAAATATTTTGAT | Promoter of TcAK2 |
| TcAK2-promoter-R-WT**^a^** | **gctagcttgatgccattcatg**TATATTTATGAAAGAAGGATACAATAATTTGA |  |
| TcAK2-promoter-F-Mt**^b^** | AATtctgctgcggagTGTAAAAAAAATTGATAAAATGCATGTT | Mutant of promoter of TcAK2 |
| TcAK2-promoter-R-Mt**^b^** | ACActccgcagcagaATTACTTTTAGCATTGTTGTTTGTATTCG |  |
| TcFOXO-F-pIZT**^a^** | **tcgaatttaaagcttggtacc**ATGGACTGCTACAAGATGAACCTCA | Overexpression of TcFOXO in Sf9 cell |
| TcFOXO-R-pIZT**^a^** | **tgctggatatctgcagaattc**TTAATGCACCCACGACGGC |  |
| TcAK1-F-pIZT**^a^** | **tcgaatttaaagcttggtacc**ATGGTTGACGCCGCAGTTT | Overexpression of TcAK1 in Sf9 cell |
| TcAK2-R-pIZT**^a^** | **tgctggatatctgcagaattc**TTACAACTCCTTTTCCATCTTGATGA |  |
| TcAK1-F-pIZT**^a^** | **tcgaatttaaagcttggtacc**ATGTCACAACACCTCAAATTATTGTATC | Overexpression of TcAK2 in Sf9 cell |
| TcAK2-R-pIZT**^a^** | **tgctggatatctgcagaattc**TCACATGCAACTCTCCATTTCAA |  |
| TcAMPKα-F | **tgtattttcagggcgccatg**ATGGGGGATGTGACCCAGG | Co-expressionof three AMPK subunits in Sf9 cells |
| TcAMPKα-R | **cttctcgacaagcttggtacc**TTAACGAGCCAACTGTATAATTAAAG |  |
| TcAMPKβ-F | **tgtattttcagggcgccatg**ATGGGCAATGCAGGCAGTG |  |
| TcAMPKβ-R | **cttctcgacaagcttggtacc**TTAAATCGGTTTATATAAAAGAGTAG |  |
| TcAMPKγ-F | **tgtattttcagggcgccatg**ATGGACTCTGATGTGTCCCTGC |  |
| TcAMPKγ-R | **cttctcgacaagcttggtacc**TTATTCACCGCCTCCCGTC |  |
| TcAMPKαT172D-F**^b^** | TTTGCGCgatAGTTGTGGTTCCCCCAATTACG | Mutant of TcAMPKα subunit |
| TcAMPKαT172D-R**^b^** | CACAACTatcGCGCAAAAACTCCCCGTCCATC |  |
| CtGFP-TcAMPKα-F | **gtgaggaactaaacc**ATGGGGGATGTGACCCAGG | Overexpression of TcAMPKα for BiFC in Sf9 |
| CtGFP-TcAMPKα-R | **ttgtctgccgtgatg**TTAACGAGCCAACTGTATAATTAAAGCGGC |  |
| TcAK1ORF-F | **gatctggttccgcgtggatcc**ATGGTTGACGCCGCAGTTT | Expression of GST fusion protein |
| TcAK1ORF-R | **tcagtcagtcacgatgcggccgc**TTACAACTCCTTTTCCATCTTGATGA |  |
| TcAK2ORF-F | **gatctggttccgcgtggatcc**ATGTCACAACACCTCAAATTATTGTATC | Expression of GST fusion protein |
| TcAK2ORF-R | **tcagtcagtcacgatgcggccgc**TCACATGCAACTCTCCATTTCAA |  |
| TcFOXOORF-F | **gatctggttccgcgtggatcc**ATGGACTGCTACAAGATGAACCTCA | Expression of GST fusion protein |
| TcFOXOORF-R | **tcagtcagtcacgatgcggccgc**TTAATGCACCCACGACGGC |  |
| TcAK1-F**^a^** | **attacggccaggcctccatgg**ATGGTTGACGCCGCAGTTT | For yeast two-hybrid |
| TcAK1-R**^a^** | **cgttaacgctttcatgcggccgc**TTACAACTCCTTTTCCATCTTGATGA |  |
| TcAK2-F^a^ | **attacggccaggcctccatgg**ATGGCAAAAGCAAAATGCCA | For yeast two-hybrid |
| TcAK2-R^a^ | **cgttaacgctttcatgcggccgc**TCACATGCAACTCTCCATTTCAA |  |
| TcAK1-T247D-F**^b^** | gacGGGGTCAACGATATCGAGAAGCGACTCCC | Mutant of TcAK1 |
| TcAK1-T247D-R**^b^** | ATATCGTTGACCCCgtcCACCAATCGACGGTAGACCTG |  |
| TcAK1-S129D-F**^b^** | CGCgacCTGGAGGGCTACCCCTTCAACCCTTG | Mutant of TcAK1 |
| TcAK1-S129D-R**^b^** | TAGCCCTCCAGgtcGCGGCCGCAGCGCACACG |  |
| TcAK2-S145D-F**^b^** | AGGTGTGGACGCgacTTGGCGGGTTATCCCTTCA | Mutant of TcAK2 |
| TcAK2-S145D-R**^b^** | AAgtcGCGTCCACACCTGATCCTGGTCGAAAC |  |
| TcAMPKα-F**^a^** | **atgttccagattacgctgga**ATGGGGGATGTGACCCAGG | For yeast two-hybrid |
| TcAMPKα-R**^a^** | **cgttgataccactgcttgga**TTAACGAGCCAACTGTATAATTAAAGC |  |
| TcAK1-CHIPq-F | AAATAGGTTGCGCCTGTCCT | For ChIP-qPCR |
| TcAK1-CHIPq-R | TTGCGACGTCTGATGGCTTA |  |
| TcAK2-CHIPq-F | TCAGTGAAAATGACACAAACGAT | For ChIP-qPCR |
| TcAK2-CHIPq-R | ACACACTAGGTACACACTTTGC |  |
| AMPKα1q-F | CTCAGTTCCTGGAGAAAGATGG | AMPKα1 RT-qPCR |
| AMPKα1q-R | CCCAGTCAATTCATGTTTGCC |  |
| AMPKα2q-F | AGTTCTACCTCGCCTCTAGTC | AMPKα2 RT-qPCR |
| AMPKα2q-R | CAATGGACATCTTGCTTTGGG |  |
| FOXO3aq-F | AGACCATCCAAGAGAACAAGC | FOXO3a RT-qPCR |
| FOXO3aq-R | GCTAAGTGAGTCCGAAGTGAG |  |
| CKBq-F | GATGAGCACAAGACCGACCT | CKB RT-qPCR |
| CKBq-R | GAGGCGAGACGGGAGTGA |  |
| GAPDHq-F | GCATGGGTCAGAAGGATTCCT | GAPDH RT-qPCR |
| GAPDHq-R | TCGTCCCAGTTGGTGACGAT |  |

**^a^The additional nucleotides complementary to the cloning site on the plasmid were in bold.**

**^b^ Sites of point mutations are underlined.**
